# Supplementary material for: ABCE1 Controls Ribosome Recycling by an Asymmetric Dynamic Conformational Equilibrium
Source: Cell Rep. 2019 Jul 16;28(3):723–734.e6. doi: 10.1016/j.celrep.2019.06.052 (PMC6656783; doi:10.1016/j.celrep.2019.06.052)
Supplement: Document S1. Figures S1–S5 and Tables S1 and S2 [file mmc1.pdf]

Cell Reports, Volume *28*

Supplemental Information

ABCE1 Controls Ribosome Recycling by an Asymmetric Dynamic Conformational Equilibrium

Giorgos Gouridis, Bianca Hetzert, Kristin Kiosze-Becker, Marijn de Boer, Holger Heinemann, Elina Nürenberg-Goloub, Thorben Cordes, and Robert Tampé

Supplemental Items

	Anisotropy R	
	Cy3B	Atto647N
Free dye	0.08 ± 0.01	0.05 ± 0.01
ds22 ruler (dsDNA/Cy3B-8bp-Atto647N)	0.13 ± 0.02	0.16 ± 0.03
ds24 ruler (dsDNA/Cy3B-18bp-Atto647N)	0.15 ± 0.03	0.17 ± 0.04
ABCE1$^{K177C/T393C}$	0.20 ± 0.03	0.22 ± 0.05
ABCE1$^{I124C/K430C}$	0.21 ± 0.04	0.23 ± 0.05

Table S1. Anisotropy values derived from ensemble measurements, Related to Figure 1 and Experimental Methods

We applied a similar strategy as described before that uses steady-state anisotropy to verify that the measurements of E* report on relative differences in inter-probe distances of donor and acceptor molecules (Gouridis et al., 2015). In this procedure, steady-state anisotropy values of protein samples and static references based on double-stranded DNA (45-bp oligonucleotides) labeled with Cy3B and Atto647N (IBA, Germany) were compared. In the dsDNA system, an increase in the separation between donor and acceptor, results in decreasing FRET efficiency, which experimentally verifies that FRET serves as a nanoscopic ruler. For DNA the anisotropy was <0.17 and <0.23 for ABCE1 (Table S1). We used a published theory (Haas et al., 1978) to estimate the relative error associated with distance determination in both dsDNA and ABCE1 when erroneously assuming a fixed dye orientation (κ2~ 2/3). Haas and co-workers provide this error as ratio r/r' of true distance r to apparent distance r'; this ratio (=uncertainty) is moderate for anisotropies r<0.3 of both dyes (Haas et al., 1978). We find r/r' <12% for dsDNA and <20% r/r' for ABCE1 variants. These results and considerations confirm that FRET can act as a nanoscopic ruler in our assays. Additional measurements of site II variant labeled with a different dye pair (Alexa555/Alexa647, ThermoFisher Scientific) confirm the three distinct conformational states at E* = 0.56 (low FRET), E* = 0.69 (intermediate FRET), E* = 0.86 (high FRET) at 50 nM of 30S, 2 mM AMP-PNP and 10 min incubation at 73 °C resembling conditions shown in Figure 3. Although the protein values are slightly elevated compared to dsDNA samples, additional control experiments with a different dye pair on ABCE1 confirm the occurrence of three different FRET states in ABCE1 with low, intermediate and high FRET efficiency as given in **Supplemental Table S2**.

	low FRET (open state)	intermediate FRET (intermediate state)	high FRET (closed state)
Site I (Cy3B/Atto647N) in free ABCE1	0.55 ± 0.04	0.64 ± 0.04	0.79 ± 0.03
Site I (Cy3B/Atto647N) in pre-SC	0.53 ± 0.07	0.65 ± 0.03	0.78 ± 0.08
Site I (Cy3B/Atto647N) in post-SC	0.50 ± 0.01	0.61 ± 0.03	0.76 ± 0.04
Site II (Cy3B/Atto647N) in free ABCE1	0.47 ± 0.02	0.60 ± 0.01	–
Site II (Cy3B/Atto647N) in pre-SC	0.49 ± 0.06	0.62 ± 0.08	0.83 ± 0.03
Site II (Cy3B/Atto647N) in post-SC	0.51 ± 0.01	0.62 ± 0.03	0.80 ± 0.05
ΔFeS Site II (Cy3B/Atto647N) in free ABCE1	–	0.61 ± 0.02	0.79 ± 0.03
ΔFeS Site I (Cy3B/Atto647N) in post-SC	0.50 ± 0.01	0.61 ± 0.02	0.75 ± 0.01
ΔFeS Site II (Cy3B/Atto647N) in post-SC	–	0.61 ± 0.01	–
Site II (Alexa555/Alexa647) in post-SC	0.56 ± 0.02	0.69 ± 0.03	0.86 ± 0.03
ds22 (dsDNA/Cy3B-8bp-Atto647N)	0.90 ± 0.04 (0.066 ± 0.001)		
ds23 (dsDNA/Cy3B-13bp-Atto647N)	0.64 ± 0.01 (0.078 ± 0.001)		
ds24 (dsDNA/Cy3B-18bp-Atto647N)	0.45 ± 0.02 (0.076 ± 0.001)		
ds27 (dsDNA/Cy3B-33bp-Atto647N)	0.17 ± 0.03 (0.061 ± 0.002)		

Table S2. Apparent FRET Values of dsDNA Standards and ABCE1 Variants with Different Labels, Related to Figure 1, 2 and Experimental Methods

It is important to note that absolute FRET values (E*) reported in this manuscript cannot be directly compared since they were not corrected for spectral crosstalk, differences in donor and acceptor fluorescence quantum yield, and different detection efficiencies of the donor and acceptor channels. Since the second tested dye-pair Alexa555/Alexa647 further has a distinct Förster radius of R_0 = 5.1 nm from Cy3B/Atto647N (R_0 = 5.5–6.5 nm) (Ploetz et al., 2016), different FRET values were obtained for 70S- and 30S-bound ABCE1. As additional controls we report mean E* and sigma-values of experiments using double-stranded DNA with fluorophores at 8 bp, 13 bp, 18 bp, or 33 bp separation. These gave us the possibility to define the standard deviation (reported into parenthesis) for static samples with different apparent FRET values (E*). The width of the distribution (standard deviation) is a variable in smFRET ALEX experiments that is (largely) independent of the molecular details of the sample, but depends on the (limited) number of photons obtained from single-molecules. The more photons that are detected from a single-molecule the smaller the standard deviation and *vice versa* (Nir et al., 2006).

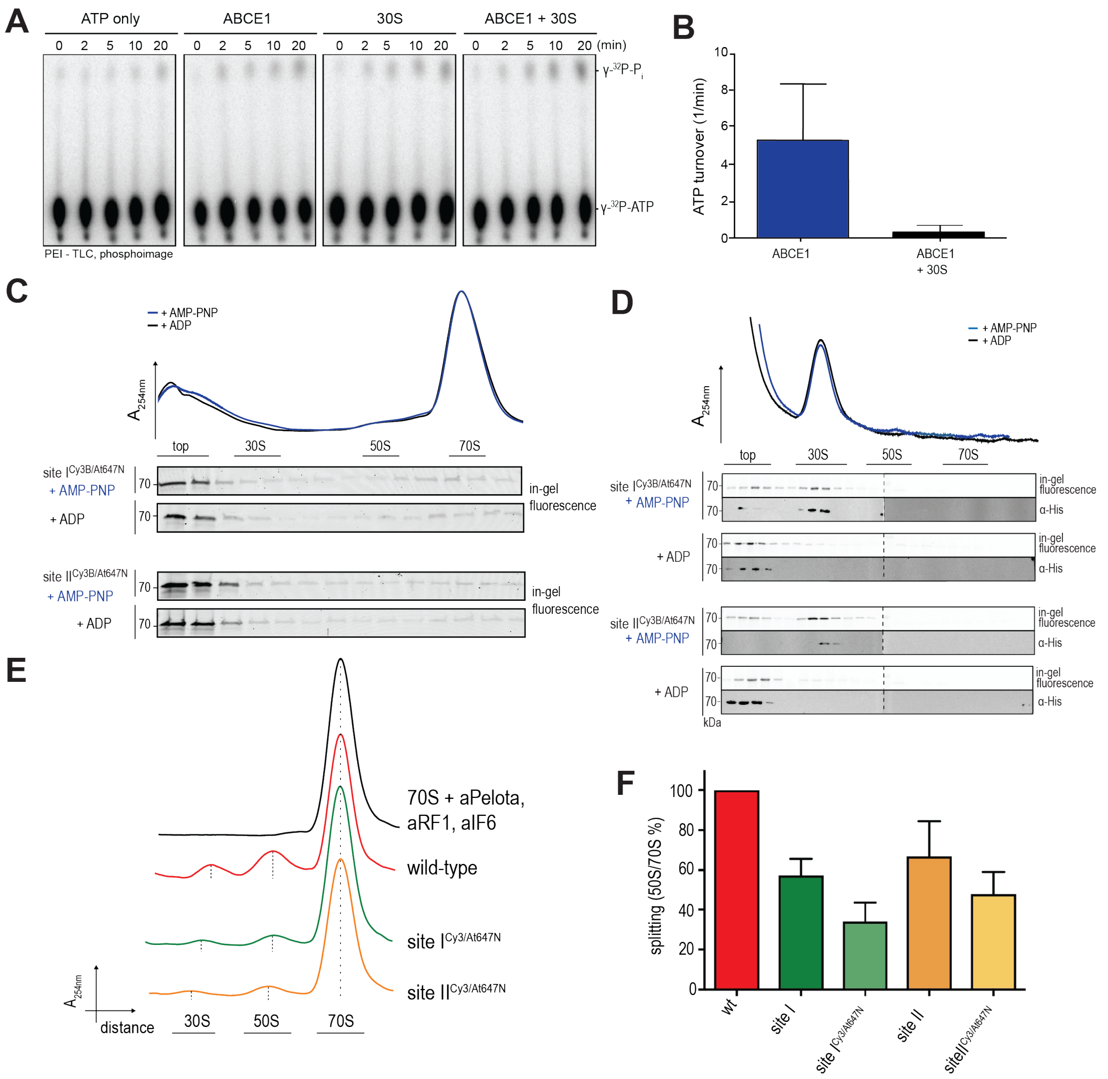

Figure S1. Formation of the Pre- and Post-SC with FRET Pair Labeled ABCE1, Related to Figure 1.

(A) Hydrolysis of γ-^{32}P-labeled ATP resulted in the formation of γ-^{32}P$_i$, which was separated by thin layer chromatography and subsequently quantified by autoradiography. Time course over 20 min. (B) ABCE1 shows no ATPase activity upon addition of 30S ribosomes. Hydrolysis of ABCE1 and ABCE1+30S were corrected for auto-hydrolysis and ABCE1+30S corrected for hydrolysis of 30S only. ATP turnover was calculated from 20 min point of time. Data derived from (B) are given as mean ± SD from three experiments. (C) AMP-PNP- and aRF1/aPelota-dependent formation of the pre-SC. A-site factors aRF1/aPelota (3 µM) and *T. celer* 70S ribosomes (3 µM) were pre-incubated for 30 min at 25 °C. Subsequently, ABCE1 (1 µM) and AMP-PNP (2 mM) were added and incubated for 1 h at 25 °C to form the complex. The specific binding of labeled site II variant to 70S ribosomes was analyzed by sucrose density centrifugation and subsequent in-gel fluorescence. (D) AMP-PNP-dependent formation of the post-SC. ABCE1 (3 µM) was incubated with 30S ribosomes (4 µM) for 10 min at 73 °C to form the post-SC. The specific binding of labeled ABCE1 variants to 30S ribosomes was analyzed by sucrose density centrifugation and subsequent in-gel fluorescence and immunoblotting. (E) Splitting of 70S (0.5 µM) by wild-type ABCE1 and site I and II variants (2 µM) with AMP-PNP (0.125 mM), aRF1/aPelota and the anti-association factor aIF6 for 15 min at 45 °C. (F) Splitting efficiency of wtABCE1 and site I and II variants. 50S/70S ratio was calculated by the peak height. Since at least 85% of ABCE1 molecules are doubly labelled, having correctly assembled FeS clusters and display a monodisperse population in size-exclusion chromatography (see text for details), we conclude that the reduction in splitting efficiency depends mainly on the cysteine mutation and minimally on fluorophore attachment. Clearly, the labelled cysteine variants (site I and II) retain high degree of functionality. The bars are normalized to the splitting activity of wtABCE1. Data represent mean ± SD from three measurements.

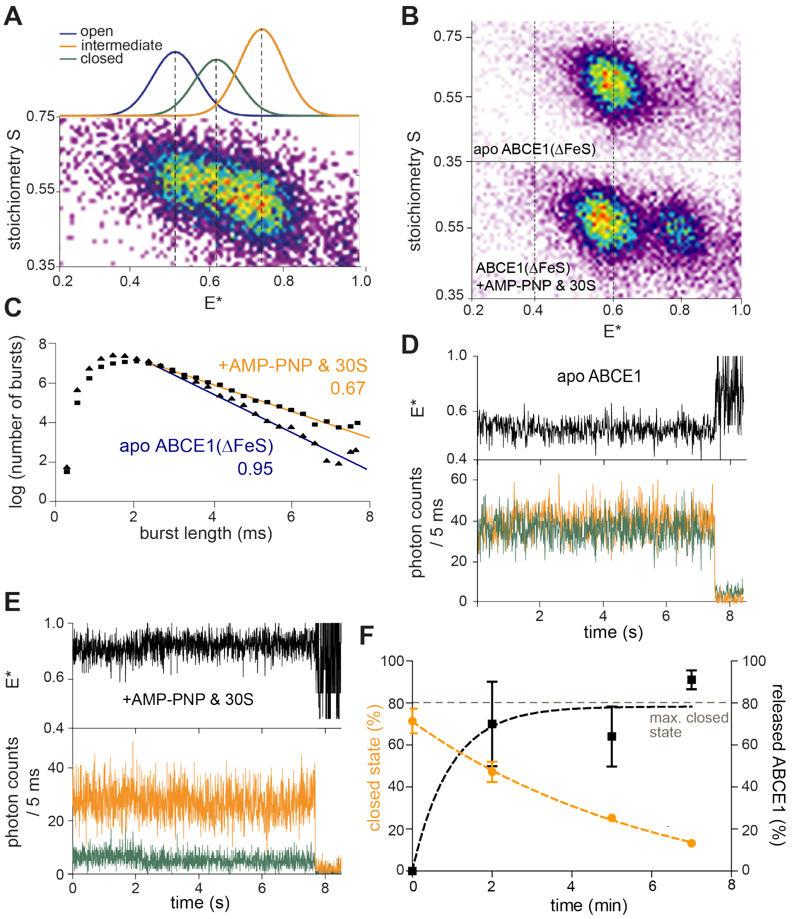

Figure S2. Conformational States of site I, ΔFeS site II and I Variant Compared to Full-length ABCE1, Related to Figure 2 and 3.

(A) 2D-ALEX histogram of site I after incubation with non-saturating concentration of 30S ribosomal subunit (25 nM) and AMPPNP (2 mM) for 10 min at 73 °C. Site I resembles an equilibrium between an open (blue, E*= 0.5), intermediate (green, E*= 0.61) and closed sate (orange, E*= 0,76). (B) 2D-ALEX analysis of ΔFeS site II variant labeled with Cy3B and Atto647N. Two different conformational states of ΔFeS site II (intermediate and closed) are observed. (C) 30S in complex with ABCE1 was investigated via an exponential fit of the burst-length distribution that indirectly probes molecular diffusion times of labeled ABCE1. The data show that also ΔFeS site II variant can bind to the ribosome and adopt the closed state. The derivation of a burst-size histogram from all molecules of one condition (apo vs. post-SC) shows that ΔFeS site II variant has reduced diffusion speed (0.67) and a small percentage of closed state (E* = 0.79) under post-splitting conditions when in complex with 30S and AMP-PNP. The intermediate value of the relative diffusion coefficient of post-SC conditions (0.67, compared to 0.53 of site II) indicates that only a subpopulation of all molecules is in complex with 30S. Specifically, we note that ABCE1 in the closed conformation (E* > 0.7) is completely 30S bound, whereas in the intermediate conformation (E* < 0.7) only a small subpopulation of ABCE1 molecules present is associated to 30S. These results demonstrate that the FeS cluster domain is not essential for conformational changes of site II or 30S-ABCE1 interaction. Nevertheless, the FeS cluster domain is essential for ribosome splitting and stable 30S association (Barthelme et al., 2011; Karcher et al., 2008). This observation is fully compatible with the fact that the closed state has the highest affinity for the 30S subunit. Since the ΔFeS site II variant-30S interaction is of short-lived nature (see also **Figure S3A/B**, compare bars 5 to 6 for ΔFeS site II or 4 to 5 for ΔFeS site II), this association could not be observed in previous experiments using sucrose density gradient centrifugation (Barthelme et al., 2011). The FeS cluster domain is required for dynamics in site II and stable ribosome interaction (Barthelme et al., 2011; Karcher et al., 2008). (D and E) Fluorescent trajectories of the immobilized site II derivative using confocal scanning microscopy analysis demonstrate the static nature of the apo (D) and the post-splitting state (E) of ABCE1 at room-temperature on the 10 s time scale. (F) The release of ABCE1 from the small ribosomal subunit (black) and its interdependence with the site I opening (orange) is depicted. Data represent mean ± SD from three measurements.

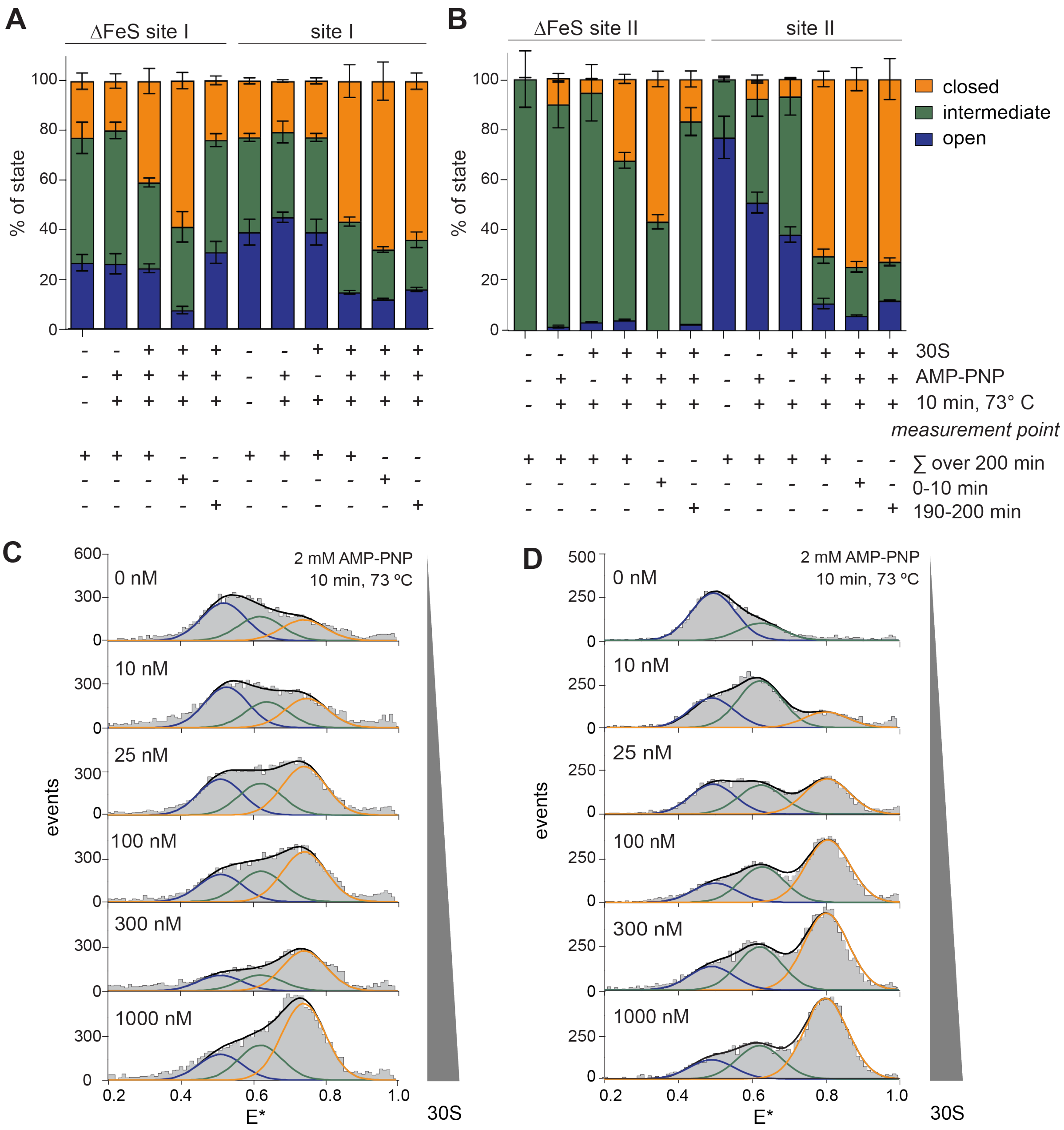

Figure S3. Conformational States of ΔFeS site II and I Variant in Comparison to Full-length Variants, Related to Figure 2, 3 and 4.

(A and B) Conformational states of ΔFeS and full-length site I and II variants under various conditions. Each measurement was performed under saturating conditions with 1 µM 30S ribosomal subunit and 2 mM nucleotide where indicated. Data represent mean ± SD from three measurements. (C and D) Conformational dynamics at site I (C) and site II (D) are probed in dependence of increasing 30S ribosomal subunit concentrations at saturating AMP-PNP concentrations (2 mM).

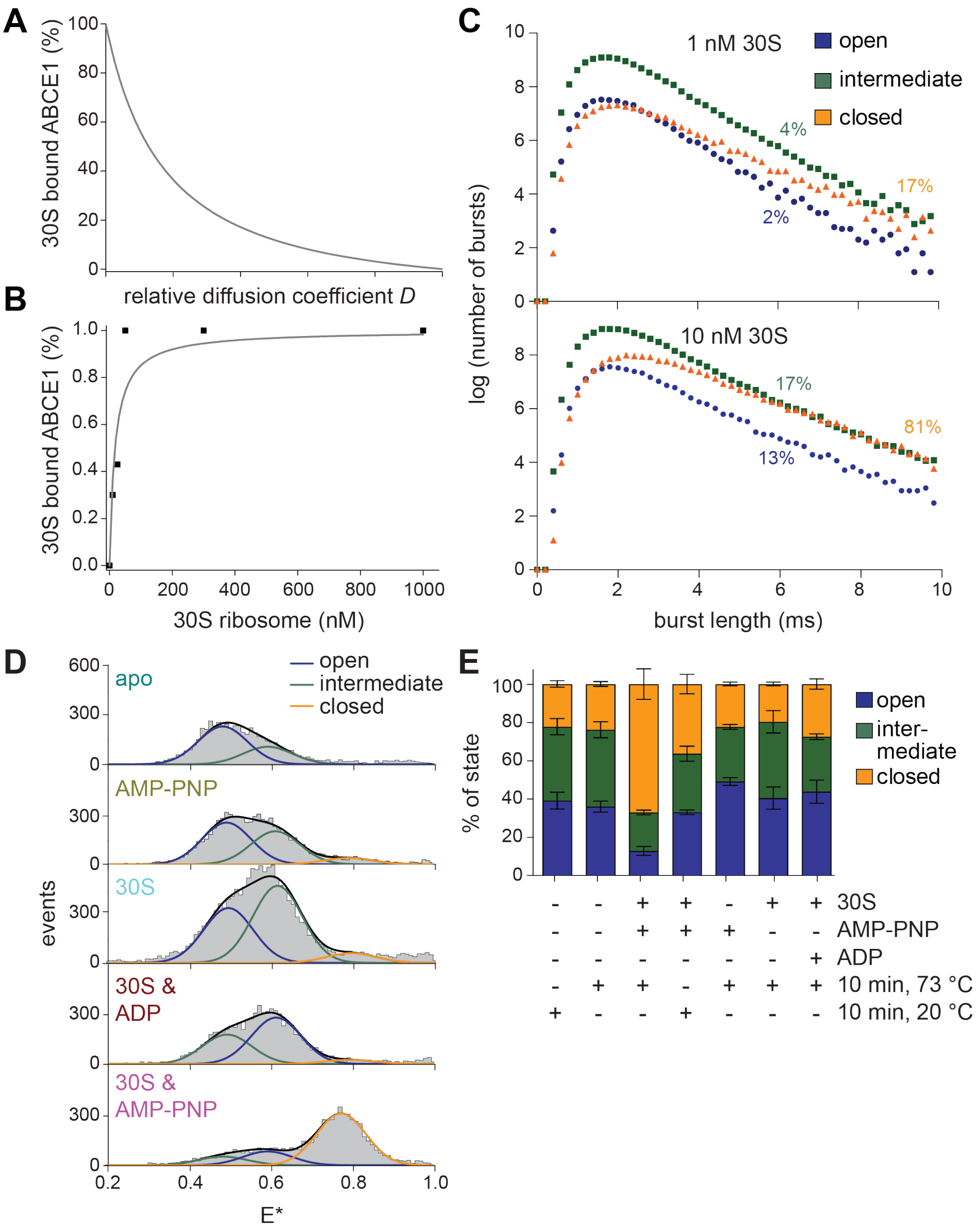

Figure S4. Different 30S Binding Affinities for the Open, Intermediate, and Closed State of ABCE1, Related to Figure 4.

(A) Relation of determined relative diffusion coefficient D (slope from exponential fits of burst-size histogram as shown in panel (C) to % of 30S-bound ABCE1 molecules (details see Materials and Methods). (B) Direct determination of 30S binding affinity for ABCE1 in the presence of 2 mM AMP-PNP using the relation between relative diffusion coefficient and 30S-bound % applying the data presented in Figure 4A and Figure S3C/D. Obtained K_D value of ~ 20 nM. (C) Experimental data to determine relative diffusion times for different ABCE1 conformational states. Here, the burst-length distribution of each FRET population (low, intermediate, high) was analyzed to derive a relative diffusion coefficient. The data shows that the high FRET state (closed) has the highest affinity since it is being >80% bound to 30S ribosome when increasing 30S from 1 nM to 10 nM, while low and intermediate FRET are still < 20% bound to 30S. (D and E) Influence of different ligands such as nucleotides and ribosomal subunits on the conformational states of site II (D) and site I (E). The experiments were performed under saturation conditions of nucleotides (2 mM) and 30S ribosomal subunits (1 µM) after 10 min incubation at 73 °C as indicated in the respective panel (D and E). Data in (E) represent mean ± SD from 3-5 measurements.

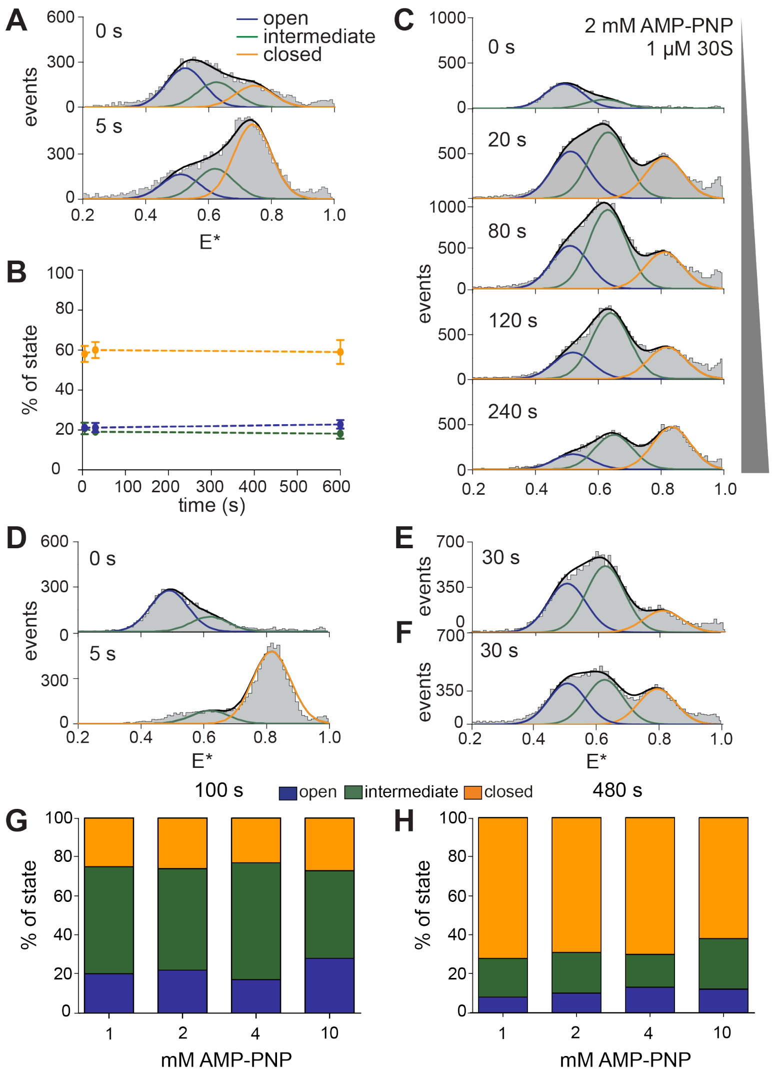

Figure S5. Elucidation of the Rate-limiting Step for Conformational Changes in Site I and the Time-dependence of the Conformational Changes in Site II, Related to Figure 4.

(A and B) The rate-determining step of conformational dynamics at site I was addressed by pre-incubated ABCE1 site I variant with AMP-PNP (2 mM) for 10 min at 73 °C (A, top panel). Subsequently, 30S subunit (1 µM) was added and confocal ALEX measurements were performed to monitor the state distribution after 5 s (A, bottom panel). Confocal ALEX measurements (A) and the changes in the distribution of the three states in site I are shown as a function of time after AMP-PNP pre-incubation (B). Data represent mean ± SD from three measurements. (C) Time dependent conformational dynamics at site II probed at saturating 30S ribosomal subunit (1 µM) and AMP-PNP concentrations (2 mM) at the indicated time points. (D-F) The rate-determining step of conformational dynamics at site II were addressed by use of pre-incubated ABCE1 site II variant either with AMP-PNP (2 mM, D), 30S (1 mM, E), or solely incubation at 73 °C (F). After incubation for 10 min at 73 °C, 30S (1 µM, D and F) or AMP-PNP (2 mM, E) were added, respectively, and confocal ALEX measurements were performed. Data represent mean ± SD from 3-5 measurements. (E and F) Controls where the pre-incubation was performed with 30S (1 mM, E) or heat only (10 min, 73 °C, F). (G and H) Conformational dynamics at site II as described for (D-F) and Fig. 4E. Pre-incubation was accomplished for 100 s (G) and 480 s (H) with 1-10 mM of AMP-PNP as the intracellular ATP concentration ranges from 1 to 10 mM and ATP hydrolysis obeys Michaelis-Menten kinetics with $K_{m,ATP} \sim 0.7$ mM. Increasing the ATP concentration from 1-10 mM increases the association rate by one order of magnitude, as this linearly correlates with the concentration. The kinetics to reach the conformational equilibria are independent of AMP-PNP concentration and require a long incubation subsequent to AMP-PNP binding.

Supplemental References:

Haas, E., Katchalski-Katzir, E., and Steinberg, I.Z. (1978). Effect of the orientation of donor and acceptor on the probability of energy transfer involving electronic transitions of mixed polarization. Biochemistry *17*, 5064-5070.
