# Supplementary material for: ABCE1 Controls Ribosome Recycling by an Asymmetric Dynamic Conformational Equilibrium
Source: Cell Rep. 2019 Jul 16;28(3):723–734.e6. doi: 10.1016/j.celrep.2019.06.052 (PMC6656783; doi:10.1016/j.celrep.2019.06.052)
Supplement: Document S2. Article plus Supplemental Information [file mmc2.pdf]

Cell Reports

Article

ABCE1 Controls Ribosome Recycling by an Asymmetric Dynamic Conformational Equilibrium

Graphical Abstract

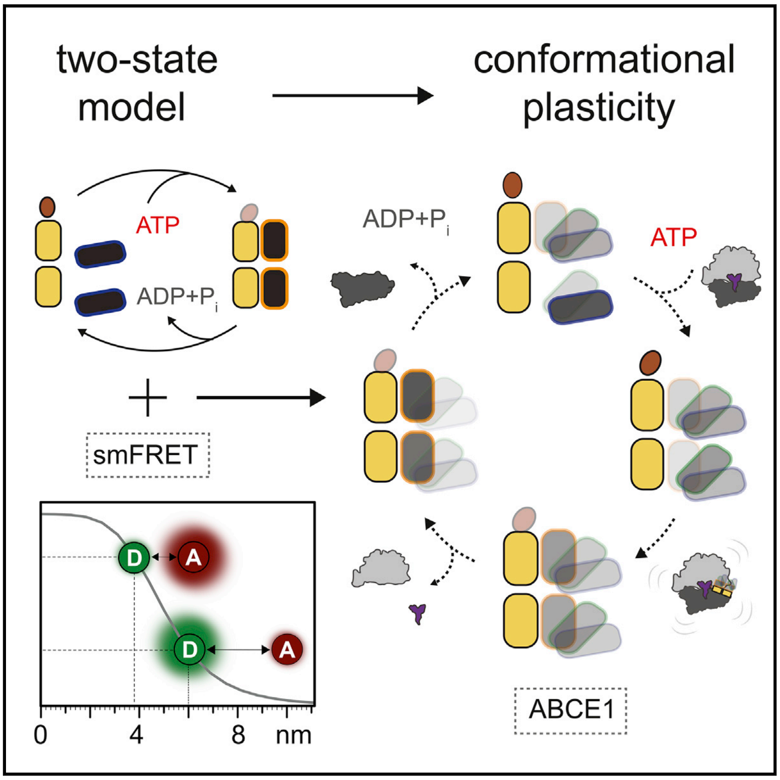

Highlights

- Both ATP sites of ABCE1 are in an asymmetric conformational equilibrium
- Each ATP site can adopt three functionally distinct conformational states
- These equilibria shift during ribosome recycling depending on interaction partners
- ATP binding, but not hydrolysis, is required for ribosome splitting

Authors

Giorgos Gouridis, Bianca Hetzert, Kristin Kiosze-Becker, ..., Elina Nürenberg-Goloub, Thorben Cordes, Robert Tampé

Correspondence

cordes@bio.lmu.de (T.C.), tampe@em.uni-frankfurt.de (R.T.)

In Brief

Gouridis et al. delineate the inner workings of ABCE1 by single-molecule FRET, demonstrating that the two asymmetric nucleotide-binding sites functionally and conformationally adopt distinct states during ribosome recycling. Unexpectedly, both sites are found in a dynamic equilibrium of conformational states governed by ribosomes, nucleotides, and release factors.

Gouridis et al., 2019, Cell Reports *28*, 723–734
July 16, 2019 © 2019 The Author(s).
https://doi.org/10.1016/j.celrep.2019.06.052

OPEN ACCESS
CellPress

ABCE1 Controls Ribosome Recycling by an Asymmetric Dynamic Conformational Equilibrium

Giorgos Gouridis,[1,2,3,5] Bianca Hetzert,[4,5] Kristin Kiosze-Becker,[4,5] Marijn de Boer,[1,5] Holger Heinemann,[4] Elina Nürenberg-Goloub,[4] Thorben Cordes,[1,2,*] and Robert Tampé[4,6,*]
[1]Molecular Microscopy Research Group, Zernike Institute for Advanced Material, University of Groningen, 9747 AG Groningen, the Netherlands
[2]Physical and Synthetic Biology, Faculty of Biology, Ludwig-Maximilians-Universität München, 82152 Planegg-Martinsried, Germany
[3]Department of Microbiology and Immunology, Rega Institute for Medical Research, Laboratory of Molecular Bacteriology, KU Leuven, 3000 Leuven, Belgium
[4]Institute of Biochemistry, Biocenter, Goethe University Frankfurt, 60438 Frankfurt a.M., Germany
[5]These authors contributed equally
[6]Lead Contact
*Correspondence: cordes@bio.lmu.de (T.C.), tampe@em.uni-frankfurt.de (R.T.)
https://doi.org/10.1016/j.celrep.2019.06.052

SUMMARY

The twin-ATPase ABCE1 has a vital function in mRNA translation by recycling terminated or stalled ribosomes. As for other functionally distinct ATP-binding cassette (ABC) proteins, the mechanochemical coupling of ATP hydrolysis to conformational changes remains elusive. Here, we use an integrated biophysical approach allowing direct observation of conformational dynamics and ribosome association of ABCE1 at the single-molecule level. Our results from FRET experiments show that the current static two-state model of ABC proteins has to be expanded because the two ATP sites of ABCE1 are in dynamic equilibrium across three distinct conformational states: open, intermediate, and closed. The interaction of ABCE1 with ribosomes influences the conformational dynamics of both ATP sites asymmetrically and creates a complex network of conformational states. Our findings suggest a paradigm shift to redefine the understanding of the mechanochemical coupling in ABC proteins: from structure-based deterministic models to dynamic-based systems.

INTRODUCTION

Ribosome recycling is an integral step of mRNA translation and surveillance at the core of protein homeostasis, ribosome-based quality control, and thus ribosome-related diseases (Jackson et al., 2012; Mills and Green, 2017; Nürenberg and Tampé, 2013). This cyclic process connects termination with initiation (Gerovac and Tampé, 2019). The ATP-binding cassette (ABC) protein ABCE1 facilitates ribosome recycling by splitting archaeal and eukaryotic ribosomes into large and small subunits (Barthelme et al., 2011; Pisarev et al., 2010; Shoemaker and Green, 2011). ABCE1 has been linked to diverse functions: innate immunity, tissue homeostasis, HIV capsid assembly, ribosome biogenesis, and translation initiation (Bisbal et al., 1995; Chen et al., 2006; Dong et al., 2004; Juszkiewicz et al., 2018; Liakath-Ali et al., 2018; Mills et al., 2016; Strunk et al., 2012; Zimmerman et al., 2002). However, given that ABCE1 is universally conserved, ribosome recycling represents the fundamental function in all organisms except bacteria (Barthelme et al., 2011; Pisarev et al., 2010; Shoemaker and Green, 2011). ABCE1 splits ribosomes either after canonical termination facilitated by release factors (e/aRF1) or after recognition of stalled and vacant ribosomes by mRNA surveillance factors (e/aPelota and Dom34 in yeast) (Barthelme et al., 2011; Pisarev et al., 2010; Shoemaker and Green, 2011; Strunk et al., 2012; van den Elzen et al., 2014).

ABCE1 belongs to the ubiquitous superfamily of ABC proteins, which use ATP binding and hydrolysis in two conserved nucleotide-binding domains (NBDs) for mechanochemical work via accessory domains (Hopfner, 2016; Locher, 2016; Thomas and Tampé, 2018). In ABCE1, two head-to-tail-oriented NBDs are linked via two composite hinge regions. Walker A and B motifs in one NBD, as well as the ABC-signature motif and D-loop in the opposing NBD, align the two ATP sites (sites I and II hereafter) to coordinate Mg(II)-ATP for a hydrolytic attack of water, which is polarized by a catalytic glutamate residue (Barthelme et al., 2011; Chen et al., 2003; Karcher et al., 2008; Smith et al., 2002). A functional and structural asymmetry of the two ATP sites has been observed in ABCE1 (Barthelme et al., 2011; Nürenberg-Goloub et al., 2018). High-resolution structures of ABCE1 suggest that ATP binding and hydrolysis cause a tweezer-like movement of the two NBDs between an open and an ATP-occluded state (Becker et al., 2012; Brown et al., 2015; Heuer et al., 2017), a mechanism that is also anticipated for ABC transporters (Chen et al., 2003; Smith et al., 2002). ABCE1 contains a unique N-terminal FeS cluster domain, harboring two diamagnetic $[4Fe\text{-}4S]^{2+}$ clusters (FeS) (Barthelme et al., 2007). In combination with an ATP-dependent motion of the NBDs, the FeS cluster domain is responsible for ribosome splitting (Barthelme et al., 2011; Becker et al., 2012; Brown et al., 2015; Heuer et al., 2017; Nürenberg-Goloub et al., 2018).

Functional and structural data provided first insights into the role of ABCE1 in ribosome recycling (Barthelme et al., 2011;

This is an open access article under the CC BY license (http://creativecommons.org/licenses/by/4.0/).

Becker et al., 2012; Heuer et al., 2017; Nürenberg-Goloub et al., 2018; Pisarev et al., 2010; Shoemaker and Green, 2011). ABCE1 binds to terminated or stalled 70/80S ribosomes in the presence of e/aRF1 or e/aPelota, establishing a pre-splitting complex (pre-SC) (Becker et al., 2012). Previous studies demonstrated that ribosome splitting depends on a mechanistic link between the FeS cluster domain and a conformational switch of the NBDs (Barthelme et al., 2011). The FeS cluster domain swings 150° out of the NBD cleft into the inter-subunit space of the ribosome, driving the subunits apart (Heuer et al., 2017). The hinge 1/2 region assists as a pivot point in closing and opening the NBD dimer. Hence, the large subunit is released and the post-splitting complex (post-SC) is available for subsequent translation initiation (Heuer et al., 2017). One key aspect is the nucleotide-dependent conformational switch of the ATP sites driving ribosome splitting. However, the conformational states of the two ATP sites in ribosome recycling have so far remained elusive. A deeper understanding of the conformational states, and particularly of the dynamics of the ATP sites, will provide essential details regarding the molecular mechanism of ABCE1 and other ABC proteins.

Therefore, we set out to study the ribosome recycling factor ABCE1 using an integrated biophysical approach that allows simultaneously the monitoring of the dynamic equilibrium of conformational states and the binding to allosteric modulators. ABCE1 from the crenarchaeon *Sulfolobus solfataricus* was chosen as a model. Because 70S ribosomes from *S. solfataricus* are intrinsically labile (Londei et al., 1986), we isolated 70S from *Thermococcus celer* (Barthelme et al., 2011; Nürenberg-Goloub et al., 2018). We used single-molecule-based Förster resonance energy transfer (smFRET) as a spectroscopic ruler to determine distance changes between two fluorescent probes and to assess conformational states and dynamics of ABCE1. Simultaneously, we monitored the diffusion properties of ABCE1 to probe its association with the 70S and 30S ribosomes.

In contrast to the deterministic two-state model of other ABC proteins [open (monomer) – closed ATP bound (dimer)], we found that in ABCE1, both sites are always in a dynamic equilibrium across three conformational states: open, intermediate, and closed. The conformational behavior of the two sites is asymmetric, allowing, for example, one site to close while the other is open. The equilibrium is biased toward the intermediate state when ABCE1 binds to 70S and toward the closed state within the post-SC. An allosteric conformational transition subsequent to ATP binding is the rate-limiting step for altering ABCE1 conformational equilibrium. Dissociation of ABCE1 from the 30S ribosomal subunit is induced by ATP hydrolysis because ADP occupancy was found to be incompatible with 30S association. This final step completes the recycling process and is followed by opening of the sites. The presented study provides unprecedented insights into the conformational landscape of ABCE1 and its dynamics at the different steps of the ribosome recycling process.

RESULTS

smFRET Monitors the Tweezer-like Movement at each ATP Site of ABCE1

To characterize the dynamic behavior of the two ATP sites of ABCE1 by smFRET, double-cysteine variants of ABCE1 were generated (Figure 1A). We used an established strategy to replace non-conserved, solvent-exposed residues by cysteines to allow site-specific labeling with organic fluorophores at strategic positions (Gouridis et al., 2015). Based on the available structural information of ADP-bound ABCE1 (Barthelme et al., 2011; Karcher et al., 2008), the variants ABCE1$^{I124C/K430C}$ and ABCE1$^{K177C/T393C}$ were created to probe the conformational states and dynamics of sites I and II, respectively. They were named site I and II variants. For fluorescent labeling, the cysteine pairs were positioned at C_α distances of approximately 5.0 nm (site I) and 4.5 nm (site II) (Figure 1A). Stochastic labeling was realized by mixing of purified ABCE1 with two fluorophores, e.g., the spectrally distinct green FRET donor (D, Cy3B) and the red FRET acceptor (A, Atto647N) (details in Method Details). By carefully optimizing the purification and labeling conditions, the eight cysteines coordinating the two FeS clusters remained intact and did not lead to detectable background labeling. In our assay, the FRET efficiency E was in the range of 0 to 1 and reports on the relative inter-probe distance (for details for deriving apparent FRET efficiency E* see Method Details), validated by steady-state fluorescence anisotropy experiments and the use of different fluorophore pairs for smFRET (compare mean FRET values of the site II variant labeled Cy3B/ATTO647N and Alexa 555/647) (Tables S1 and S2).

Expression conditions and the purification strategy, using metal affinity, anion exchange, and size exclusion chromatography, were optimized for all ABCE1 variants (Figures 1B and 1C; Method Details). Protein absorbance (280 nm) and fluorescent intensities (Cy3B, 559 nm; Atto647N, 645 nm) indicated high degrees of labeling (>85%). The characteristic absorbance of the FeS clusters was monitored at 410 nm, and only the labeled ABCE1 samples with correctly assembled FeS clusters were further analyzed (Barthelme et al., 2007). The activity of FRET-labeled ABCE1 variants was verified by determining the basal ATP hydrolysis, which was similar to the wild type (turnover ≈ 5 ATP/min) (Figure 1D; Figures S1A and S1B). Nucleotide-dependent formation of the pre-SC and post-SC, as well as ribosome splitting, was confirmed for all labeled ABCE1 variants (Figures S1C–S1F).

To derive insights into the conformational dynamics of ABCE1, we aimed to probe the conformational states of sites I and II at different steps of the splitting cycle that were previously assigned to distinct conformational states by chemical cross-linking, mass spectrometry, and cryoelectron microscopy (cryo-EM) studies (Becker et al., 2012; Brown et al., 2015; Heuer et al., 2017; Kiosze-Becker et al., 2016). The goal of our present study was to arrest intermediates of the recycling process in the form of stable nucleotide-free or nucleotide-bound states using ADP or the non-hydrolysable ATP analog AMP-imidodiphosphate (PNP) and appropriate conditions for ribosome association. The conformational dynamics of ABCE1, which provide the basis for a holistic understanding of its molecular mechanism, have not been assessed to date. Because *S. solfataricus* ABCE1 operates at physiological temperatures of 70°C–80°C, the conformational dynamics of ABCE1 from this organism can be locked at any time by rapid cooling to 4°C (Barthelme et al., 2011). Thus, any population distribution determined by smFRET represents a static snapshot of the respective conformational equilibrium at physiological temperatures.

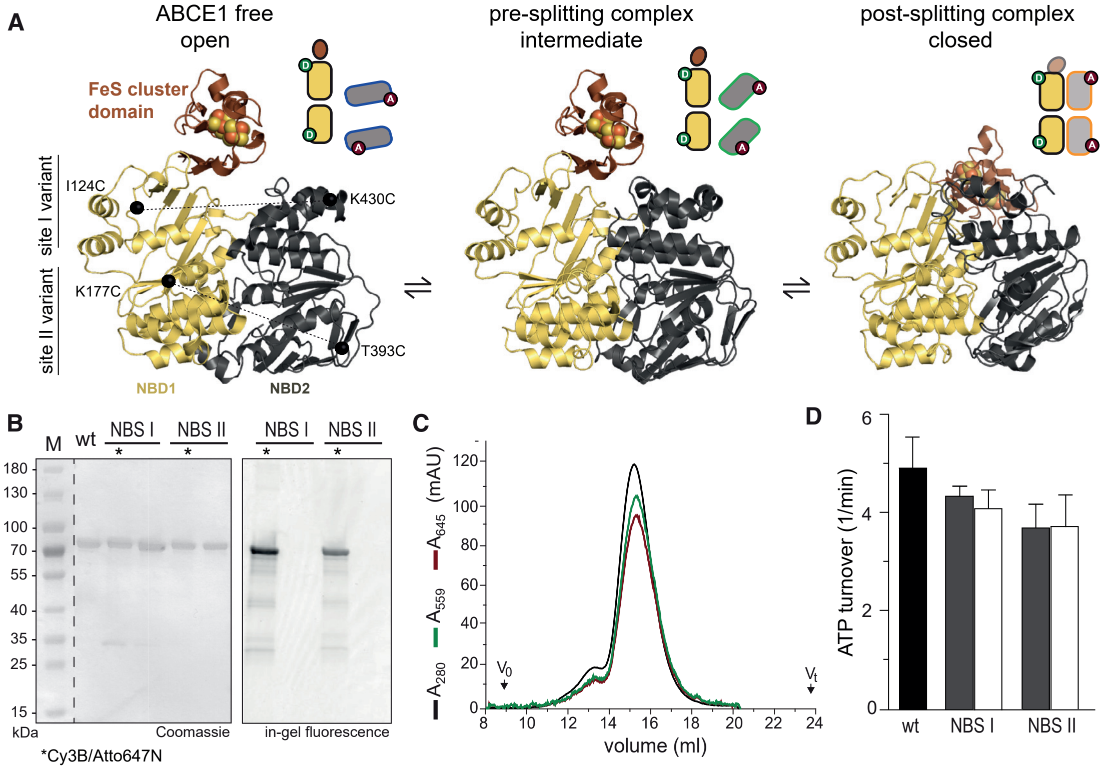

Figure 1. Biochemical Function of FRET Pair-Labeled ABCE1

(A) Double-cysteine variants probing the conformational states of site I (ABCE1$^{I124C/K430C}$) or site II (ABCE1$^{K177C/T393C}$) via smFRET are depicted on the crystal structures of ABCE1 (Barthelme et al., 2011; Karcher et al., 2008). Middle: cryo-EM structure of the ribosome-bound (pre-splitting complex) intermediate state of ABCE1 (Becker et al., 2012). Right: cryo-EM structure of the closed (ATP bound) state bound to the small ribosomal subunit (post-splitting complex) (Heuer et al., 2017).

(B) ABCE1 wild type and variants were purified to homogeneity by metal affinity and anion exchange chromatography. SDS-PAGE (12.5%, Coomassie and in-gel fluorescence). Donor and acceptor fluorophores are illustrated as D and A, respectively.

(C) FRET pair-labeled ABCE1 variants were analyzed by fluorescence-based size-exclusion chromatography (SEC) and subsequently used for smFRET.

(D) ATPase activity of ABCE1 before (gray) and after (white) fluorescence labeling. Data represent mean ± SD from three independent experiments.

Both ATP Sites Show Distinct Equilibria of Three Conformational States

We used microsecond alternating laser excitation (µs-ALEX) (Hohlbein et al., 2014; Kapanidis et al., 2004) of freely diffusing molecules to map the conformational equilibria of the two ATP sites. Here, fluorescently labeled ABCE1 enters the excitation volume of a confocal microscope for milliseconds, allowing determination of the apparent FRET efficiency E* and stoichiometry S (details in Method Details; data in Figures 2A and 2C and in Figures S2A and S2B). To probe the association of fluorescently labeled ABCE1 to the 70S or 30S ribosome, we relied on the large increase in molecular mass and correlated slower diffusion to 70S- or 30S-bound ABCE1 molecules as independently observable (Figures 2B and 2D; Figure S2C; see Method Details for data analysis). We first analyzed the conformational dynamics of site I in free ABCE1. Site I is found in a low FRET state with a distribution centered at E* ≈ 0.60 (Figure 2A, upper panel, black line). Because this distribution (1) is asymmetric and (2) exceeds shot-noise expectations (for a comparison of single versus multiple states, see data in Figure S2A versus Figure S2B, upper panel), it was fitted with a mixture model of Gaussian distributions, for which details are provided in the STAR Methods section. The smFRET data for site I are best described by the existence of three conformational states in free ABCE1: a low (blue fit), an intermediate (green fit), and a high (orange fit) FRET state with a relative abundance of approximately 50%, 30%, and 20%, respectively (represented also with the appropriate transparency in the cartoon). Pre-SCs purified by sucrose density gradient centrifugation (denoted pre-SC throughout the manuscript; 70S·aRF1/aPelota·ABCE1·AMP-PNP) exhibit no significant shift of the state distribution for site I (Figure 2A, middle panel; Method Details). In the post-SC (30S·ABCE1·AMP-PNP), site I still displays three distinct conformational states (Figure 2A, bottom panel). In contrast, site II only shows a low and an intermediate FRET state for free ABCE1 (Figure 2C, upper panel). Within the pre-SC, the state distribution is shifted primarily toward the intermediate state (E* ≈ 0.63) with a small but significant population of a high FRET state (Figure 2C, middle panel). Post-SC formation shifts the FRET distribution in site II to >80% toward the high FRET state (E* ≈ 0.84).

The relation between FRET efficiency and relative inter-probe distances (Table S2) allowed us to link the low FRET state to the

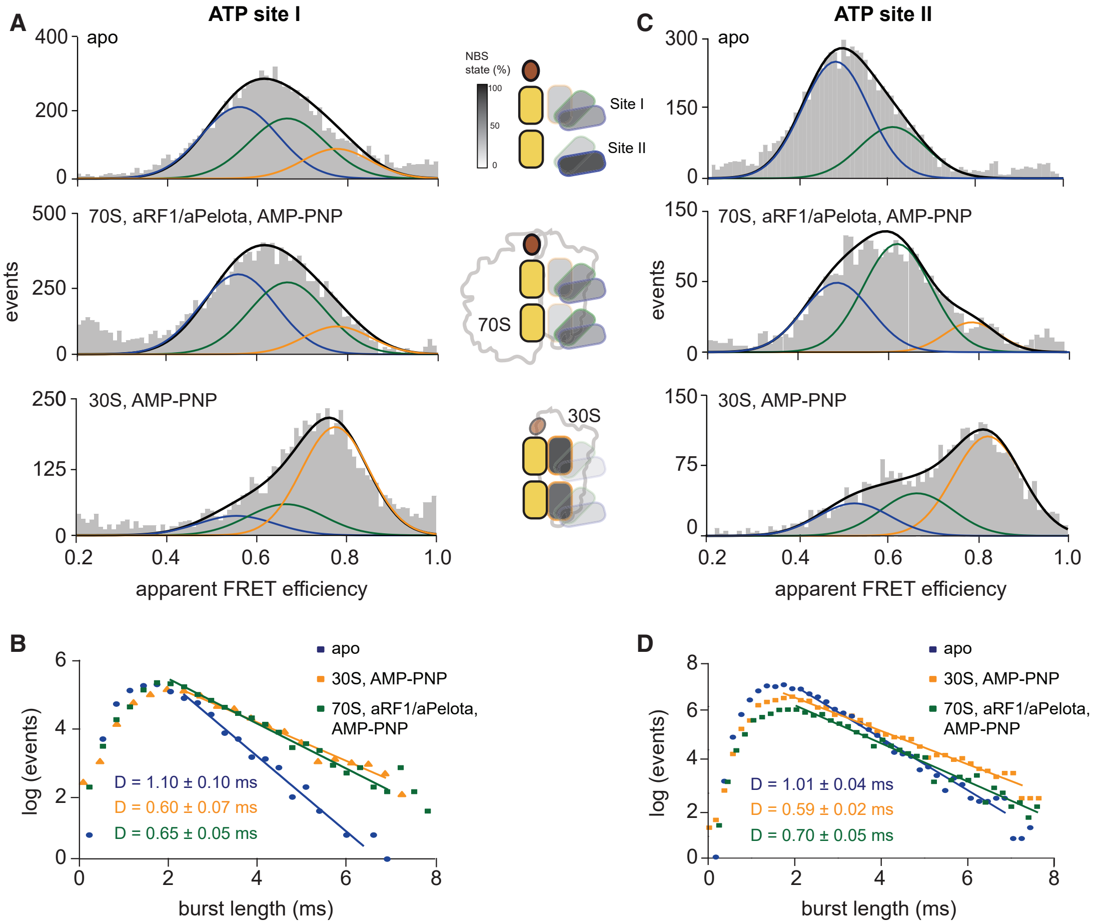

Figure 2. Conformational States of ATP Sites I and II of ABCE1 Revealed by ALEX-Based smFRET

(A and C) 2D-ALEX histogram of site I (A) and site II (C) variants labeled with Cy3B and Atto647N reveals three conformational states (open, intermediate, and closed). 2D-ALEX histogram of free ABCE1 (top panel); ABCE1 with AMP-PNP, 70S, and aRF1/aPelota (middle panel); and AMP-PNP and 30S (bottom panel). Interaction partners (3 μM 70S and aRF1/aPelota or 1 μM 30S ribosome, 2 mM AMP-PNP) affect these states. For clarity, only the double-labeled donor-acceptor species are shown. Cartoons depict the percentage of each state in the conformational equilibrium, with different transparencies as indicated.

(B and D) Burst-size histogram of site I (B) and II (D) variants of free ABCE1 and the pre-splitting state and post-splitting state of ABCE1 reveals the diffusion properties of the complexes and allows determination of a relative diffusion coefficient D by fitting the histogram to Equation 1 (Method Details). Errors indicate 95% confidence interval.

open state of the ATP sites as revealed by X-ray crystallography (Barthelme et al., 2011; Karcher et al., 2008), the intermediate FRET state to the intermediate state (Becker et al., 2012; Brown et al., 2015), and the high FRET state to the closed state (Heuer et al., 2017). The existence of an intermediate state was confirmed by analysis of the site II variant lacking the FeS cluster domain (ΔFeS site II) (Figure S2B, upper panel), because this variant uniquely adopts the intermediate conformation in free ABCE1 (mean FRET value of 0.61 ± 0.01) (Table S2). Furthermore, we confirmed the existence of the same three conformational states for a different fluorophore pair (Alexa 555/647) (Table S2), supporting their biological relevance.

To exclude conformational dynamics in the (sub-)second timescale and, in this way, to validate the conformational arrest of ABCE1 at low temperature, we performed confocal scanning experiments. For this, labeled ABCE1 was site-specifically immobilized at a C-terminal His_6 tag via an anti-His antibody. The data show that both the open and the closed states of site II are fully static because no interconversion across the states could be observed (Figures S2D and S2E).

To confirm ribosome association of ABCE1 for the pre-SC and post-SC in different conformational states, we analyzed the respective burst length distributions (details in Method Details). Taking the distinct hydrodynamic volumes of free and ribosome-associated ABCE1 into account, we directly related the burst length to the diffusion properties of different FRET populations (Figures 2B and 2D; for details, see Method Details). Relative diffusion constants D (in units of reciprocal milliseconds [ms^{-1}]) were derived by fitting the tail of the burst length distribution (>2 ms) with an exponential distribution (Figures 2B and 2D, colored fits). Free ABCE1 displays fast diffusion (D = 1.10 ± 0.10 ms^{-1}). In contrast, binding to the 70S ribosome in the presence of aRF1/aPelota, previously confirmed by sucrose density gradient centrifugation (Figure S1C), results in significantly slower diffusion

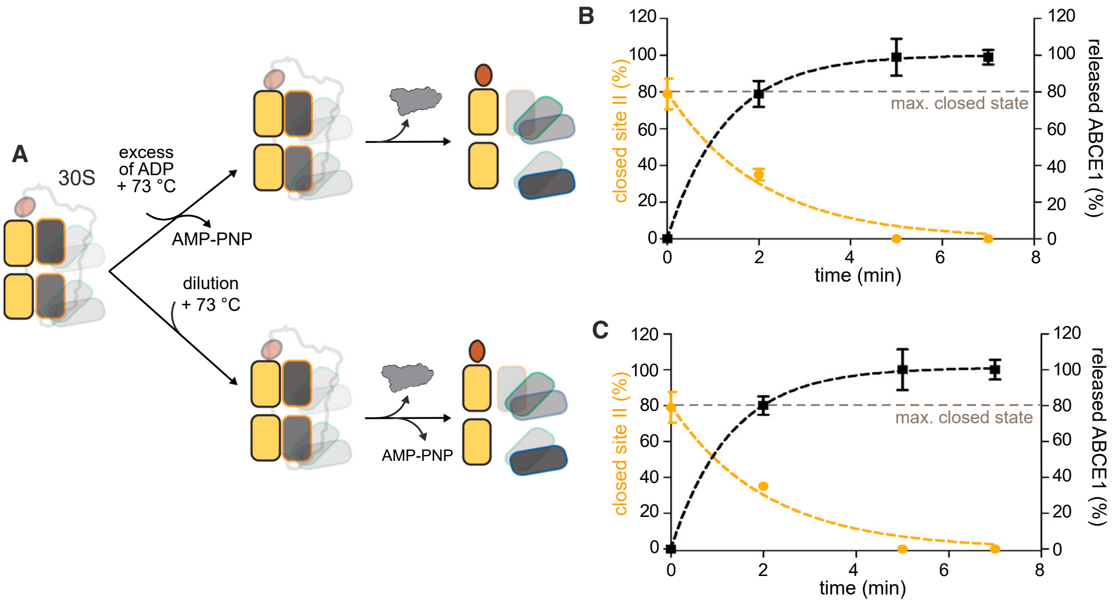

Figure 3. Release of ABCE1 from the Small Ribosomal Subunit Is Followed by Site II Opening

(A) Cartoon summarizing the experimental settings.

(B and C) Release of ABCE1 from the small ribosomal subunit (black) occurring (B) only at the physiological temperature (70°C–80°C) and simultaneous ADP competition or (C) by 30S and AMP-PNP dilution at the indicated time points (manual mixing didn't allow earlier time points), and its interdependence with site II opening (orange). Data represent mean ± SD from four independent experiments.

(D = 0.65 ± 0.05 ms^{-1}). The increase in the relative diffusion constant is consistent with the Stokes-Einstein equation ($D \propto m^{-1/3}$) of a spherical particle of mass m having a diffusion constant D (free ABCE1 has a mass of 70 kDa, and ABCE1 in complex with the 70S ribosome has a mass of 2.5 MDa). Similarly, ABCE1 in the post-SC (30S·ABCE1·AMP-PNP) displays a slight decrease in the diffusion constant (D = 0.60 ± 0.07 ms^{-1}). Analyses of the burst length distributions indicate full binding of both site I and site II variants to either 70S or 30S ribosomes (Figures 2B and 2D). Altogether, we are able to demonstrate that both ATP sites of ABCE1 dynamically sample three distinct conformational states at every step of the splitting cycle (free, pre-SC, and post-SC; i.e., open, intermediate, and closed) with a pronounced asymmetry. For example, site I of free ABCE1 can sample the closed state, while site II only acquires open and intermediate states.

The FeS Cluster Domain Modulates the Conformational Equilibrium of ATP Site II

To address the effect of the FeS cluster domain on the ABCE1 conformational equilibrium and its association with the ribosome, we created derivatives of ABCE1 with an N-terminal truncation. ΔFeS ABCE1 has a drastic effect on site II dynamics by stabilizing a unique intermediate state (Figure S2B, upper panel). Formation of the post-SC can also be achieved by ΔFeS ABCE1 (Figures S2B, bottom panel, and S2C). Site II fails to acquire the open state in the truncated derivative at all steps (free, pre-SC, and post-SC), while the dynamics of site I remain unaffected and are almost identical to those observed with the full-length ABCE1 (Figures S3A and S3B). Because the post-SC obtained with ΔFeS ABCE1 is short lived (Figure S3A, compare column 5 with column 4; Figure S3B, compare column 6 with column 5), dissociating in the hour timescale at room temperature, this transient association was not observed by sucrose density gradient centrifugation (Barthelme et al., 2011). The FeS cluster domain is required for dynamics in site II and stable ribosome interaction (Barthelme et al., 2011; Heuer et al., 2017; Karcher et al., 2008) that depends on the conformational states of site II (Nürenberg-Goloub et al., 2018). The dissimilar effects of the FeS cluster domain on the two sites are in line with the asymmetry between the sites.

Dissociation of the Post-SC Precedes the Opening of the Two ATP Sites

We next addressed the release of ABCE1 from the post-SC because this event terminates the recycling process. Dissociation can be induced solely at a physiological temperature (73°C) either by competition with ADP or dilution to nullify the association rate (Figure 3A; for details, see Method Details). Our data demonstrate that at 73°C, ABCE1 release occurs within minutes and shifts the equilibria of both site I and site II more toward the open state (Figures 3B and 3C; Figure S2F). After release, the conformational equilibrium resembles the free ABCE1 state. The 30S-ABCE1 dissociation kinetics appear to be slightly faster than changes in the conformational equilibrium of ABCE1 (Figures 3B and 3C, right versus left y axis), suggesting that opening of the ATP sites is an event following post-SC dissociation. Release of ABCE1 can only be achieved at 73°C, while the post-SC is locked at a low temperature (Figures S3A and S3B). The ATP turnover of ABCE1 in the presence of 30S (Figure S1B) is inhibited (Nürenberg-Goloub et al., 2018), indicating that an allosteric trigger is needed to induce hydrolysis in the post-SC. Our observations demonstrate that at physiological temperatures, ABCE1 continuously samples distinct

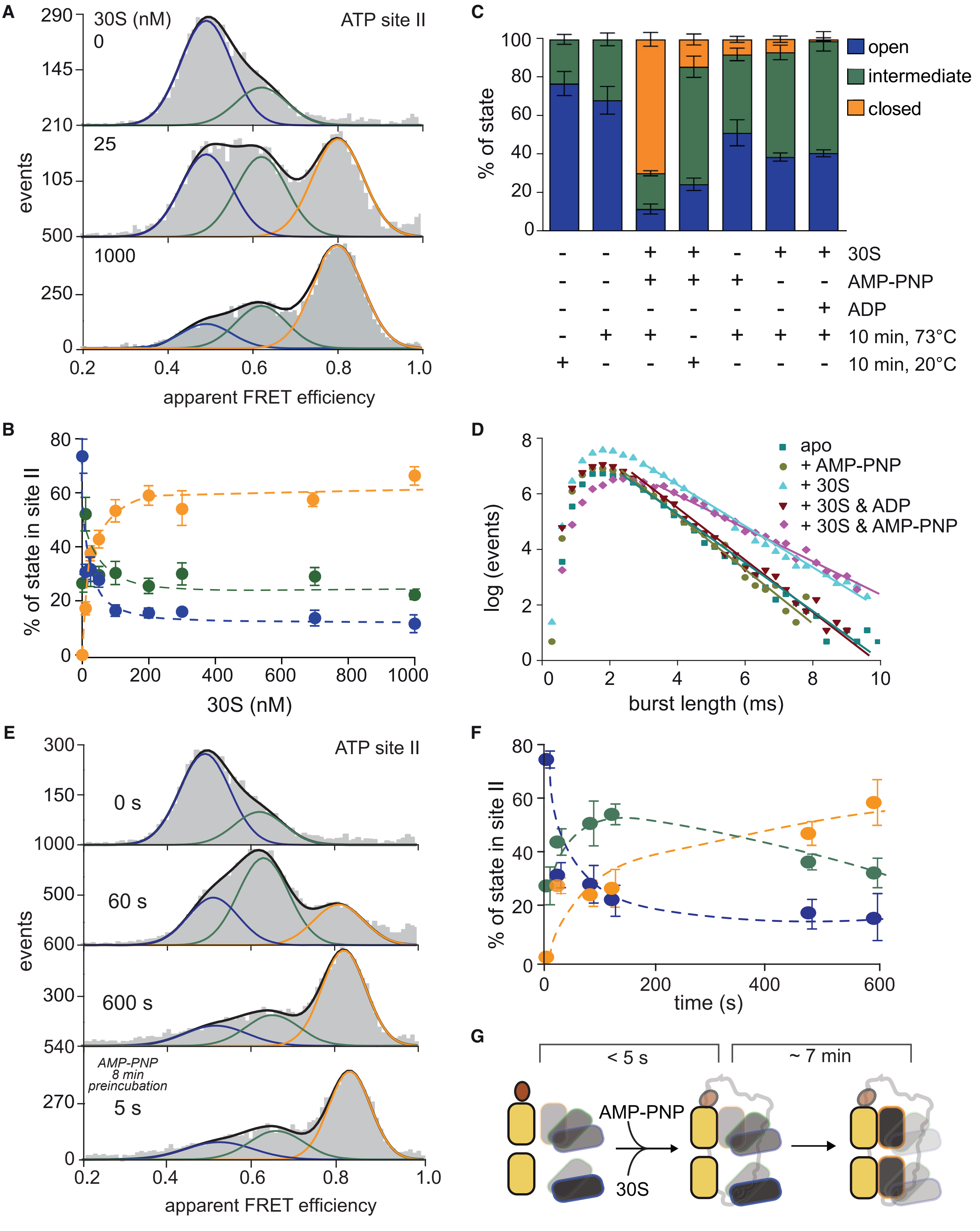

(legend on next page)

conformations even while engaged with the small ribosomal subunit (Figure 2), allowing exchange of nucleotides for release of ABCE1 from 30S ribosomes.

Ribosome and Nucleotide Binding Control the Conformational Equilibrium of the Two ATP Sites

To understand the molecular mechanisms of ABCE1, in light of its diverse functions, we investigated the key factors that can influence its conformational landscape. In more detail, we aimed to unravel the requirements for changing the conformational equilibrium in both ABCE1 sites via ligands (ribosomal subunits and nucleotides) and physicochemical parameters (temperature). Unfortunately, ABCE1 in the pre-SC is difficult to examine because this is a short-lived, unstable intermediate state of the recycling cycle (Becker et al., 2012; Nürenberg-Goloub et al., 2018) that can only be stabilized by using high magnesium concentrations and low temperatures (Figure 2; Method Details). These factors render the pre-SC of ABCE1 unsuitable for systematic investigations regarding the influence of factors on conformational dynamics. In contrast, the post-SC can be arrested as a stable complex by AMP-PNP or mutants that cannot hydrolyze ATP (Barthelme et al., 2011; Kiosze-Becker et al., 2016; Nürenberg-Goloub et al., 2018). ABCE1 undergoes the largest changes in its conformational equilibrium between the post-SC and the free state (Figure 2). Consequently, the conformational dynamics of ABCE1 were probed during incubation with 30S ribosomes and nucleotides (AMP-PNP or ADP) at the physiological temperature (73°C).

Increasing 30S concentrations (Figure 4A; Figures S3C and S3D) shift the conformational equilibrium of both sites toward the closed state (high FRET, orange) at the expense of the open state (low FRET, blue; Figure 4B). In site I, the population of the intermediate state remains constant over the entire concentration range of added 30S ribosomes, while it displays a maximum in site II at 10 nM of 30S (Figure 4B; Figures S3C and S3D). The gradual titration of 30S ribosomes and the percentage of 30S-bound ABCE1 at each concentration (Figures S4A and S4B; Method Details) yield a K_D value $\approx$ 20 nM for the ABCE1-30S interaction. At saturating conditions, all ABCE1 molecules are bound to 30S ribosomes (Figure S4B), but all three conformational states (open, intermediate, and closed) are still observed in both ATP sites (Figures 2, 4A, and 4B). Because the conformational dynamics in site II are expected to influence ribosome association (Nürenberg-Goloub et al., 2018), we addressed this interaction strength for each state of site II. Therefore, we estimated the percentage of 30S-associated ABCE1 for each state at 1 or 10 nM of 30S. This required long measurements to acquire sufficient statistics (>25,000 events) to distinguish the degrees of 30S binding (Figure S4C). As expected, the highest binding affinity was found for the closed state, followed by the intermediate and open states. These data highlight the functional differences across the conformational states, simultaneously confirming that they exist side by side. Although unlikely, given the trends in the data, labeling of ABCE1 could influence the conformational landscape of the ATP sites.

The Interplay among Conformational Transitions, Nucleotide Binding, and Ribosome Association

In the post-SC, site I and II equilibria display the most dramatic shift toward the closed state (Figures 2 and 4). Only smaller changes were detected after sole binding to different nucleotides (ADP or AMP-PNP) or 30S in the absence of nucleotides (Figure 4C; Figures S4D and S4E), even at a physiological temperature (73°C). At a low, non-physiological temperature, site II acquires a minimal percentage of the closed state in the presence of 30S and AMP-PNP (Figure 4C versus Figure S4E). An analysis of diffusion times reveals that binding of ABCE1 to 30S is possible with or without AMP-PNP but not in the presence of ADP (Figure 4D). Changes in the conformational equilibrium resulting from different available ligands and conditions again differ in both sites. For example, site II undergoes significant conformational transitions at low temperature when interacting with AMP-PNP, 30S, and ADP, while site I minimally shifts its equilibrium under such conditions (compare Figure 4C with Figure S4E and Figure S3A with Figure S3B). Furthermore, as discussed, the population of the intermediate conformation is distinct for both sites when titrating 30S ribosomes (Figure 4B; Figures S3C and S3D). These results are in agreement and support our interpretations that the conformational asymmetry in the two sites sets the base for their functional asymmetry and distinct roles during ribosome recycling (Barthelme et al., 2011; Nürenberg-Goloub et al., 2018). In addition, the high-affinity ABCE1-30S interaction (K_D $\approx$ 20 nM) is lost as soon as ABCE1 binds ADP, which is in accordance with our dissociation experiments (Figure 3). All findings provide striking evidence for the essential role of ATP hydrolysis in triggering post-SC dissociation for a new round of translation.

Because the most pronounced shifts are in the conformational equilibrium between the free ABCE1 and the post-SC, we identify key requirements to alter the conformational equilibrium of ABCE1. Our data show that ABCE1 is dynamic but manifests extreme conformational changes when transiting between the free state and the post-SC state. As established in the previous sections (Figure 4; Figures S3 and S4), three components are required to shift the ATP sites into the closed state: (1) binding to 30S, (2) binding of AMP-PNP, and (3) physiological

Figure 4. Conformational Dynamics at Site II Depend on Binding Partners and Ligands

(A) Conformational equilibrium (73°C, 10 min) of the different ABCE1 site II states after incubation with saturating AMP-PNP concentrations (2 mM) and two representative 30S concentrations, as indicated.

(B) As in (A) with the full range of 30S concentrations. At each concentration, the percentage of every state was determined and plotted.

(C) Influence of different ligands such as nucleotides and ribosomal subunits on the conformational dynamics at site II. Measurements were performed under saturation conditions of nucleotides (2 mM) and 30S ribosomal subunits (1 μM) after 10 min of incubation at 73°C.

(D) Burst length distribution of the different conditions (as in C) were analyzed as previously described (Figures 2B and 2D).

(E) Time course experiment after incubation with saturating AMP-PNP (2 mM) and 30S (1 μM) concentrations at the representative points in time, as indicated (top 3 panels). Bottom panel: same experiment after pre-incubation with AMP-PNP (73°C, 2 mM, 8 min).

(F) As in (E), with the full range of points in time. At each point in time, the percentage of every state was determined and plotted.

(G) Cartoon summarizing the findings of (E) and (F). Data represent mean ± SD from 3–5 independent experiments.

temperature (73°C) (Figures 4E and 4F; Figure S5). Incubation of ABCE1 at 73°C with AMP-PNP in the absence of 30S induces no (or minor) changes in the conformational equilibrium with respect to the state of both sites in free ABCE1 (Figure 4C; Figure S4E). However, pre-incubation for 10 min at 73°C in the presence of AMP-PNP and subsequent addition of 30S are sufficient to reach equilibria within only 5 s (Figure 4E, bottom panel; Figures S5A and S5B). No other experimental conditions, e.g., pre-incubation with 30S ribosomes before the addition of AMP-PNP (compare Figure S5E with Figure S5A) or sole heating before addition of 30S and AMP-PNP (compare Figure S5F with Figure S5A) showed similarly fast changes of the conformational equilibrium. These findings indicate that an allosteric conformational transition subsequent to AMP-PNP binding is the rate-limiting step for conformational changes, not 30S ribosome binding (Figure 4G). Because the observed kinetics do not depend on AMP-PNP concentration (Figure S5G), nucleotide binding cannot be rate limiting per se. The formation of the closed state is even slower when ABCE1 is first incubated with 30S ribosomes compared with simultaneous addition of 30S ribosomes and AMP-PNP (compare Figure S5E with Figures S5F and S5C, displaying the complete time course of 30S binding).

The dissociation rate caused by AMP-PNP depletion (0.5 min^{-1}) (Figure 3), together with the dissociation constant ($K_D \approx$ 20 nM) (Figure S4B), allows an estimation of the association rate of ABCE1 with 30S ribosomes of about $4 \cdot 10^5$ M^{-1} s^{-1}. In the presence of 1 μM 30S, ABCE1 binding to 30S ribosomes takes on average only 2.5 s. Thus, against our expectations, the rate-limiting step for site closing is not ribosome association but rather a conformational change triggered by AMP-PNP binding. Such conformational changes cause critical local structural rearrangements that do not involve large-scale domain motions, which are not detected in our smFRET analysis (Figure 4C; Figure S4E).

DISCUSSION

In this study, we elucidated the conformational dynamics and plasticity of the ribosome recycling factor ABCE1 with single-molecule resolution. Previous structural studies provided important insights into the arrangement of the recycling factor ABCE1 in its free form (Barthelme et al., 2011; Karcher et al., 2008), in the pre-SC (Becker et al., 2012; Brown et al., 2015), and in the post-SC (Heuer et al., 2017) and therefore formed the framework for mechanistic investigations. However, the fundamentals of a true structure-function relationship for ABCE1 remained elusive because of the complex conformational landscape of the protein. Only the most abundant conformational states of the ABCE1 sites can be stabilized under crystallization conditions or selected using the picking algorithms in cryo-EM analysis. While this information is essential, only additional knowledge on the conformational dynamics of ABCE1 (i.e., by single-molecule resolution procedures; Lerner et al., 2018) can reveal all underlying principles of the mechanism of this molecular motor.

Our data offer insights on different factors influencing the conformational equilibria of ABCE1 and will allow the decoding of the role of specific conformational states in ribosome splitting. With our approach, engagement and disengagement of the NBDs were shown to proceed over three distinct conformational states of the two ATP sites that are in asymmetric dynamic equilibrium: open, intermediate, and closed. This dynamic equilibrium is an intrinsic property of ABCE1. Based on our data, we anticipate that all three states fulfill different functions to precisely regulate the multi-step process of ribosome recycling. We also observed conformational asymmetry in the two ATP sites. Assuming independence of both ATP sites, ABCE1 can potentially acquire nine (3^2) distinct conformers considering sites I and II. However, the current smFRET assay does not allow the conformational states of both ATP sites to be probed in parallel. This model still disregards the additional complexity arising from FeS cluster domain motions. We speculate that the high conformational plasticity of ABCE1 revealed in this study is essential to regulate ribosome recycling and to conduct all other distinct functions (Gerovac and Tampé, 2019).

Knowledge regarding the complexity of the ABCE1 conformational landscape, in combination with previous functional and structural information, allows us to propose a working model for the mechanism of ABCE1 in ribosome recycling (Figure 5): In free ABCE1 (step 1), site II predominately populates the open state, while site I is extremely plastic, adopting all three conformations. Binding of ABCE1 to 70S ribosomes, facilitated by A-site factors e/aRF1 or e/aPelota, leads to the formation of the pre-SC (step 2). Here, the conformational equilibrium of site II is shifted toward the intermediate state, consistent with cryo-EM structures (Becker et al., 2012; Brown et al., 2015; Shao et al., 2016). The ABCE1-70S interaction solely affects the conformational dynamics of site II, an observation that is in line with the specific requirements of site II acting like a switch to probe for proper 70S interaction (Nürenberg-Goloub et al., 2018). The pre-SC is a short-lived, unstable complex (Becker et al., 2012; Nürenberg-Goloub et al., 2018) that can only be stabilized by high magnesium concentrations and low temperatures (Figure 2; Method Details). At physiological conditions, ATP (AMP-PNP)-loaded ABCE1 mediates splitting of splitting-competent ribosomes (step 2 → 3). In this process, ATP binding to ABCE1 (not hydrolysis) induces ribosome splitting, as demonstrated under single-turnover conditions (Barthelme et al., 2011; Heuer et al., 2017; Nürenberg-Goloub et al., 2018). In contrast, under multiple-turnover conditions, addition of AMP-PNP reduces ribosome splitting by ABCE1 (Pisarev et al., 2010; Shoemaker and Green, 2011). Our data indicate that ribosome splitting coincides with acquisition of the closed state (Figure 4) that requires ATP occlusion (Figure 4C) but not ATP hydrolysis.

Our study demonstrates that the rate-limiting step in switching the conformational equilibria of ABCE1 to the closed conformation, in which the nucleotide is occluded and the FeS cluster domain rearranges to promote splitting (Heuer et al., 2017; Kiosze-Becker et al., 2016), is an allosteric event subsequent to ATP binding, which occurs on the minute timescale (Figure 4G). Moreover, ATP hydrolysis and ADP generation, which also take place on the minute timescale (Barthelme et al., 2011), cause immediate release of ABCE1 from the small ribosomal subunit (Figure 3A). Altogether, we conclude that ribosome splitting (Figure 5, step 2 → 3) and non-productive ATP hydrolysis (Figure 5, step 2 → 1) are two parallel, competing pathways in the *in vitro* system, which is in line with previously proposed models (Gerovac

OPEN ACCESS
CellPress

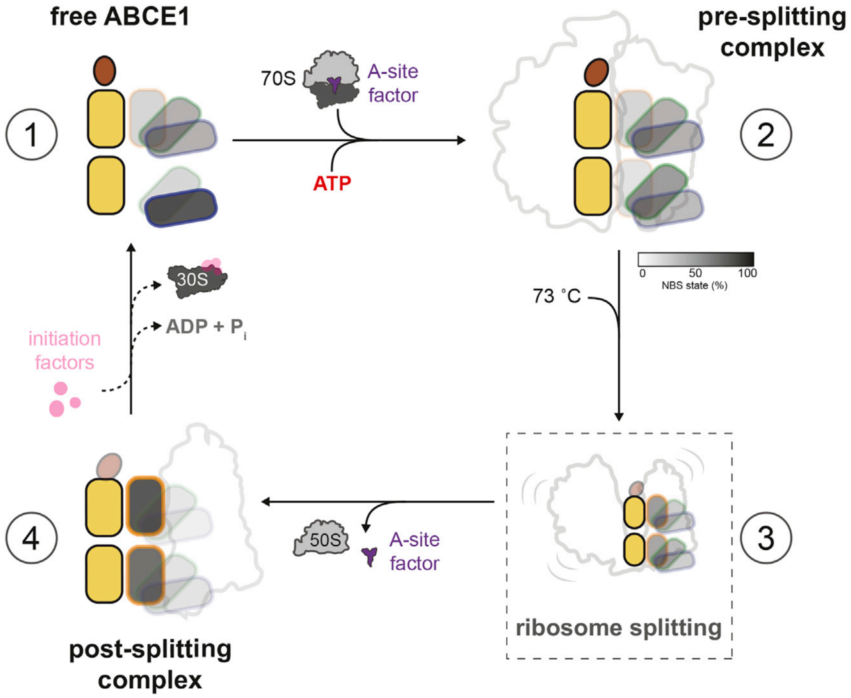

Figure 5. Dynamics of ABCE1 in Ribosome Recycling

Step 1: free ABCE1 sites are in dynamic equilibrium across three states (open, intermediate, and closed) but predominantly found in the open conformation. ABCE1 displays basal ATPase activity of 5 ATP per minute (see Figure 1D). Step 2: complex with the terminated 70S is mediated by the A-site factors e/aRF1 or e/aPelota and ATP binding. Upon formation of the pre-SC, only site II shifts to the intermediate state, as indicated, and the FeS cluster domain moves toward NBD2 (intermediate; see Figure 2A). Step 3: during splitting, ATP binding and incubation at a physiological temperature trigger the two sites to close, and the FeS cluster domain is repositioned 150° on 30S in the post-SC (closed; see Figures 2A and 2C). Here, either the FeS cluster domain pushes the A-site factor farther into the cleft between the subunits or the domain splits the subunits apart. Step 4: after splitting, bound ABCE1 can build a platform for re-initiation (Heuer et al., 2017). Acquisition of the ADP state triggers dissociation of the post-SC to initiate a new round. ABCE1 is highly dynamic, being at every condition in equilibrium across the indicated conformational states. The percentages of open, intermediate, and closed states for the 2 sites have been experimentally determined for steps 1, 2, and 4. The unstable short-lived step 3 is anticipated to have intermediate values of steps 3 and 4.

and Tampé, 2019; Nürenberg-Goloub et al., 2018). This idea is consistent with the observation that both events are triggered and related to the acquisition of the closed state of ABCE1 (Figure 4). Whenever the occluded state is formed, splitting can take place (Figure 5, step 2 → 3). When ATP hydrolysis precedes splitting, as in the case of splitting incompetent ribosomes, ABCE1 can be released from the 70S ribosome and, upon ATP binding, associates with other ribosomes (Figure 5, step 2 → 1). We also speculate that ribosome splitting (Figure 5, step 3) might be an additional trigger for ATP hydrolysis. Thus, step 3 may directly decay into free ABCE1 and, if ATP hydrolysis is well timed and occurs just after step 3, dissociated ribosmes can be formed. We anticipate that in the *in vivo* situation, the conformational transitions of ABCE1 are coupled to its regulatory role in ribosome recycling within the diverse cellular pathways (Gerovac and Tampé, 2019; Nürenberg-Goloub et al., 2018).

If during detachment of the large ribosomal subunit ABCE1 remains loaded with ATP, the post-SC is formed (Figure 5, step 3 → 4); alternatively, it could be formed via binding of free 30S to ABCE1 because of their high-affinity interaction (Figure 5, step 1 → 4). In the post-SC, ATP hydrolysis is strongly inhibited (Figure S1B) and the conformational equilibrium is biased toward the occluded ATP-bound state. ATP-loaded ABCE1 has a high affinity for 30S (Figure S4B), and the post-SC is stable: at a physiological temperature, it dissociates only by addition of an excess of ADP or an enormous dilution of the sample (Figure 3A). At a low temperature, the post-SC is stable for hours (Figures S3A and S3B). Evidently, *in vivo*, the post-SC provides a potential platform for the recruitment of initiation factors (Heuer et al., 2017) or at least requires additional factors for dissociation of ABCE1 and 30S. Consistently, ATP hydrolysis in the post-SC might be allosterically controlled by binding of initiation factors to 30S and allosteric crosstalk through 16S rRNA helix 44 within the 30S ribosome. This hypothesis is supported by the FeS cluster domain, as well as archaeal IF1A, IF1, and IF2γ, binding to helix 44 (Coureux et al., 2016). Because ABCE1-ADP does not associate with 30S ribosomes (Figures 3B, 3C, and 4D), the small subunit detaches after ATP hydrolysis and the conformational equilibrium of ABCE1 is progressively shifted toward its free state (Figure 5, step 4 → 1). Hence, ABCE1 is liberated to initiate a new round of ribosome splitting.

For better understanding of the mechanisms of ABCE1, a detailed analysis of the interdependence and conformational crosstalk between site I and site II is needed. In that respect, functional and structural asymmetry was identified as one key element for the mechanism of ABCE1 (Barthelme et al., 2011; Nürenberg-Goloub et al., 2018) and has been discussed for other ABC proteins (Hohl et al., 2012; Mishra et al., 2014; Zutz et al., 2011). In addition, by means of X-ray crystallography and molecular dynamics simulation, allosteric crosstalk of both sites was reported for other ABC-type proteins upon substrate binding (George and Jones, 2012; Grossmann et al., 2014; Hohl et al., 2012). For ABCE1, a simultaneous analysis of site I and II functional variants via multicolor FRET experiments (Ha, 2014; Lee et al., 2007, 2010) would be desirable to address all questions related to correlated movement. Our smFRET studies give first indications on the relevance of the FeS cluster domain for shaping the conformational landscape of the ATP sites and its influence on the affinity of ABCE1 for ribosomal subunits (Figures S2B, S2C, S3A and S3B). In addition, the direct observation of the FeS cluster domain movement in the pre- and post-SC would be of prime interest to gain a complete picture of the splitting

process and thus reveal the complexity of possible ABCE1 states. For future work, however, limitations in specific labeling of the FeS cluster domain, which contains eight highly conserved cysteines coordinating the FeS cluster, have to be overcome to enable direct visualization of the domain movement.

Although our findings offer insights into the conformational states and dynamics of ABCE1 and thus enhance our understanding on ribosome recycling, they have significant and general implications for the molecular mechanisms of ABC proteins. According to the current model of mechanochemical coupling of ABC transporters, the NBDs are linked to the transmembrane domains to coordinate conformational changes required for alternating access using ATP-driven cycles of monomerization (equivalent to the open state formed after ATP hydrolysis) and dimerization (equivalent to the closed state formed after ATP binding). The two-state model system is thus created by interaction of the NBDs with ATP and ADP/inorganic phosphate (P_i). Studies examining protein dynamics at the single-molecule level confirmed the two-state model system in different transport-related NBDs (Husada et al., 2018; Liu et al., 2018; Yang et al., 2018). Analogously for ABCE1, it has been postulated that its ATPase activity drives processes like ribosome remodeling, chromosome condensation, or membrane transport (Nürenberg and Tampé, 2013; Rees et al., 2009; van der Does and Tampé, 2004). This hypothesis was based on functional and structural data obtained from analyses by X-ray crystallography and cryo-EM showing that free ABCE1, ATP-bound, and ADP-bound states were solved in distinct conformations. Our findings provide important insights into the conformational states of the highly conserved ABC-type NBDs and show no such tight correlation. It is evident that these conclusions can only arise from single-molecule approaches (Mickler et al., 2009) that do not average heterogeneous mixtures and do not rely on homogeneous preparations and states as required for structural analysis. In summary, ABCE1 shows loose coupling between nucleotide occupancy and NBD conformational states. We thus speculate that this feature is related to the diverse roles and easy modulation of ABCE1 by various partners to conduct distinct biological functions.

STAR★METHODS

Detailed methods are provided in the online version of this paper and include the following:

- KEY RESOURCES TABLE
- LEAD CONTACT AND MATERIALS AVAILABILITY
- EXPERIMENTAL MODEL AND SUBJECT DETAILS
  - Bacterial strains
- METHOD DETAILS
  - Plasmids
  - Protein purification
  - Ribosome purification
  - Fluorescent labeling of ABCE1
  - Malachite Green ATPase assay
  - Radioactive ATPase assay
  - Formation of the pre- and post-splitting complex and ribosome splitting assay
  - Sample preparation for smFRET and ALEX
  - Anisotropy measurements and verification of the FRET-ruler character
  - Single-molecule fluorescence microscopy and ALEX
  - ALEX data analysis
  - Confocal scanning microscopy and data analysis
- QUANTIFICATION AND STATISTICAL ANALYSIS
- DATA AND CODE AVAILABILITY

SUPPLEMENTAL INFORMATION

Supplemental Information can be found online at https://doi.org/10.1016/j.celrep.2019.06.052.

ACKNOWLEDGMENTS

This work was funded by the German Research Council (grant SFB 902 to R.T.), the Cluster of Excellence-Macromolecular Complexes (support to R.T.), the European Research Council (ERC Starting Grant 638536 to T.C.), and NWO (VENI grant 722.012.012 to G.G.). T.C. was further supported by the Center of Nanoscience Munich (CeNS), the German Research Council within GRK2062 (project C03) and SFB 863 (project A13), LMUexcellent, and the Cluster of Excellence-Center for Integrated Protein Science Munich (CiPSM). G.G. acknowledges the EMBO long-term fellowship program (ALF 47-2012) and financial support by the Zernike Institute for Advanced Materials. G.G. is a Rega foundation postdoctoral fellow. We thank M. Gerovac and S. Trowitzsch for critical discussions and M. Roelfes for providing support to establish new data analysis tools. We thank Harald Huber, Centre of Microbiology & Archaea, University of Regensburg, for providing *T. celer* cell pellets. Correspondence and requests for materials should be addressed to T.C. (cordes@bio.lmu.de) and R.T. (tampe@em.uni-frankfurt.de).

AUTHOR CONTRIBUTIONS

R.T. initiated the project. G.G., B.H., K.K.-B., T.C., and R.T. conceived the study. T.C. and R.T. coordinated the project. B.H. and K.K.-B. prepared all protein samples and conducted biochemical experiments. H.H. and E.N.-G. contributed to the biochemical assays. G.G. established the labeling protocols and designed single-molecule assays. G.G., B.H., K.K.-B., and M.d.B. performed single-molecule experiments. G.G. and M.d.B. analyzed the smFRET data. M.d.B. contributed new tools for data analysis. All authors discussed results, interpreted data, and commented on the manuscript. G.G., B.H., T.C., and R.T. wrote the manuscript.

DECLARATION OF INTERESTS

The authors declare no competing interests.

Received: September 1, 2016
Revised: February 4, 2019
Accepted: June 14, 2019
Published: July 16, 2019

SUPPORTING CITATIONS

The following reference appears in the Supplemental Information: Haas et al. (1978).

REFERENCES

Albers, S.V., Szabó, Z., and Driessen, A.J.M. (2003). Archaeal homolog of bacterial type IV prepilin signal peptidases with broad substrate specificity. J. Bacteriol. *185*, 3918–3925.

Barthelme, D., Scheele, U., Dinkelaker, S., Janoschka, A., Macmillan, F., Albers, S.V., Driessen, A.J., Stagni, M.S., Bill, E., Meyer-Klaucke, W., et al. (2007).

Structural organization of essential iron-sulfur clusters in the evolutionarily highly conserved ATP-binding cassette protein ABCE1. J. Biol. Chem. *282*, 14598–14607.

Barthelme, D., Dinkelaker, S., Albers, S.V., Londei, P., Ermler, U., and Tampé, R. (2011). Ribosome recycling depends on a mechanistic link between the FeS cluster domain and a conformational switch of the twin-ATPase ABCE1. Proc. Natl. Acad. Sci. USA *108*, 3228–3233.

Baykov, A.A., Evtushenko, O.A., and Avaeva, S.M. (1988). A malachite green procedure for orthophosphate determination and its use in alkaline phosphatase-based enzyme immunoassay. Anal. Biochem. *171*, 266–270.

Becker, T., Franckenberg, S., Wickles, S., Shoemaker, C.J., Anger, A.M., Armache, J.P., Sieber, H., Ungewickell, C., Berninghausen, O., Daberkow, I., et al. (2012). Structural basis of highly conserved ribosome recycling in eukaryotes and archaea. Nature *482*, 501–506.

Bisbal, C., Martinand, C., Silhol, M., Lebleu, B., and Salehzada, T. (1995). Cloning and characterization of a RNAse L inhibitor. A new component of the interferon-regulated 2-5A pathway. J. Biol. Chem. *270*, 13308–13317.

Brown, A., Shao, S., Murray, J., Hegde, R.S., and Ramakrishnan, V. (2015). Structural basis for stop codon recognition in eukaryotes. Nature *524*, 493–496.

Chen, J., Lu, G., Lin, J., Davidson, A.L., and Quiocho, F.A. (2003). A tweezers-like motion of the ATP-binding cassette dimer in an ABC transport cycle. Mol. Cell *12*, 651–661.

Chen, Z.Q., Dong, J., Ishimura, A., Daar, I., Hinnebusch, A.G., and Dean, M. (2006). The essential vertebrate ABCE1 protein interacts with eukaryotic initiation factors. J. Biol. Chem. *281*, 7452–7457.

Coureux, P.D., Lazennec-Schurdevin, C., Monestier, A., Larquet, E., Cladière, L., Klaholz, B.P., Schmitt, E., and Mechulam, Y. (2016). Cryo-EM study of start codon selection during archaeal translation initiation. Nat. Commun. *7*, 13366.

Dong, J., Lai, R., Nielsen, K., Fekete, C.A., Qiu, H., and Hinnebusch, A.G. (2004). The essential ATP-binding cassette protein RLI1 functions in translation by promoting preinitiation complex assembly. J. Biol. Chem. *279*, 42157–42168.

Eggeling, C., Berger, S., Brand, L., Fries, J.R., Schaffer, J., Volkmer, A., and Seidel, C.A. (2001). Data registration and selective single-molecule analysis using multi-parameter fluorescence detection. J. Biotechnol. *86*, 163–180.

George, A.M., and Jones, P.M. (2012). Perspectives on the structure-function of ABC transporters: the Switch and Constant Contact models. Prog. Biophys. Mol. Biol. *109*, 95–107.

Gerovac, M., and Tampé, R. (2019). Control of mRNA Translation by Versatile ATP-Driven Machines. Trends Biochem. Sci. *44*, 167–180.

Gouridis, G., Schuurman-Wolters, G.K., Ploetz, E., Husada, F., Vietrov, R., de Boer, M., Cordes, T., and Poolman, B. (2015). Conformational dynamics in substrate-binding domains influences transport in the ABC importer GlnPQ. Nat. Struct. Mol. Biol. *22*, 57–64.

Grossmann, N., Vakkasoglu, A.S., Hulpke, S., Abele, R., Gaudet, R., and Tampé, R. (2014). Mechanistic determinants of the directionality and energetics of active export by a heterodimeric ABC transporter. Nat. Commun. *5*, 5419.

Ha, T. (2014). Single-molecule methods leap ahead. Nat. Methods *11*, 1015–1018.

Heuer, A., Gerovac, M., Schmidt, C., Trowitzsch, S., Preis, A., Kötter, P., Berninghausen, O., Becker, T., Beckmann, R., and Tampé, R. (2017). Structure of the 40S-ABCE1 post-splitting complex in ribosome recycling and translation initiation. Nat. Struct. Mol. Biol. *24*, 453–460.

Hohl, M., Briand, C., Grütter, M.G., and Seeger, M.A. (2012). Crystal structure of a heterodimeric ABC transporter in its inward-facing conformation. Nat. Struct. Mol. Biol. *19*, 395–402.

Hohlbein, J., Craggs, T.D., and Cordes, T. (2014). Alternating-laser excitation: single-molecule FRET and beyond. Chem. Soc. Rev. *43*, 1156–1171.

Hopfner, K.P. (2016). Invited review: Architectures and mechanisms of ATP binding cassette proteins. Biopolymers *105*, 492–504.

Husada, F., Gouridis, G., Vietrov, R., Schuurman-Wolters, G.K., Ploetz, E., de Boer, M., Poolman, B., and Cordes, T. (2015). Watching conformational dynamics of ABC transporters with single-molecule tools. Biochem. Soc. Trans. *43*, 1041–1047.

Husada, F., Bountra, K., Tassis, K., de Boer, M., Romano, M., Rebuffat, S., Beis, K., and Cordes, T. (2018). Conformational dynamics of the ABC transporter McjD seen by single-molecule FRET. EMBO J. *37*, e100056.

Jackson, R.J., Hellen, C.U., and Pestova, T.V. (2012). Termination and post-termination events in eukaryotic translation. Adv. Protein Chem. Struct. Biol. *86*, 45–93.

Juszkiewicz, S., Chandrasekaran, V., Lin, Z., Kraatz, S., Ramakrishnan, V., and Hegde, R.S. (2018). ZNF598 Is a Quality Control Sensor of Collided Ribosomes. Mol. Cell *72*, 469–481.e7.

Kapanidis, A.N., Lee, N.K., Laurence, T.A., Doose, S., Margeat, E., and Weiss, S. (2004). Fluorescence-aided molecule sorting: analysis of structure and interactions by alternating-laser excitation of single molecules. Proc. Natl. Acad. Sci. USA *101*, 8936–8941.

Karcher, A., Schele, A., and Hopfner, K.P. (2008). X-ray structure of the complete ABC enzyme ABCE1 from Pyrococcus abyssi. J. Biol. Chem. *283*, 7962–7971.

Kiosze-Becker, K., Ori, A., Gerovac, M., Heuer, A., Nürenberg-Goloub, E., Rashid, U.J., Becker, T., Beckmann, R., Beck, M., and Tampé, R. (2016). Structure of the ribosome post-recycling complex probed by chemical cross-linking and mass spectrometry. Nat. Commun. *7*, 13248.

Ko, D.S., Sauer, M., Nord, S., Müller, R., and Wolfrum, J. (1997). Determination of the diffusion coefficient of dye in solution at single molecule level. Chem. Phys. Lett. *269*, 54–58.

Lee, N.K., Kapanidis, A.N., Koh, H.R., Korlann, Y., Ho, S.O., Kim, Y., Gassman, N., Kim, S.K., and Weiss, S. (2007). Three-color alternating-laser excitation of single molecules: monitoring multiple interactions and distances. Biophys. J. *92*, 303–312.

Lee, J., Lee, S., Ragunathan, K., Joo, C., Ha, T., and Hohng, S. (2010). Single-molecule four-color FRET. Angew. Chem. Int. Ed. Engl. *49*, 9922–9925.

Lerner, E., Cordes, T., Ingargiola, A., Alhadid, Y., Chung, S., Michalet, X., and Weiss, S. (2018). Toward dynamic structural biology: Two decades of single-molecule Forster resonance energy transfer. Science *359*, eaan1133.

Leshin, J.A., Rakauskaitė, R., Dinman, J.D., and Meskauskas, A. (2010). Enhanced purity, activity and structural integrity of yeast ribosomes purified using a general chromatographic method. RNA Biol. *7*, 354–360.

Liakath-Ali, K., Mills, E.W., Sequeira, I., Lichtenberger, B.M., Pisco, A.O., Sipilä, K.H., Mishra, A., Yoshikawa, H., Wu, C.C.-C., Ly, T., et al. (2018). An evolutionarily conserved ribosome-rescue pathway maintains epidermal homeostasis. Nature *556*, 376–380.

Liu, Y., Liu, Y., He, L., Zhao, Y., and Zhang, X.C. (2018). Single-molecule fluorescence studies on the conformational change of the ABC transporter MsbA. Biophys. Rep. *4*, 153–165.

Locher, K.P. (2016). Mechanistic diversity in ATP-binding cassette (ABC) transporters. Nat. Struct. Mol. Biol. *23*, 487–493.

Londei, P., Altamura, S., Cammarano, P., and Petrucci, L. (1986). Differential features of ribosomes and of poly(U)-programmed cell-free systems derived from sulphur-dependent archaebacterial species. Eur. J. Biochem. *157*, 455–462.

Mickler, M., Hessling, M., Ratzke, C., Buchner, J., and Hugel, T. (2009). The large conformational changes of Hsp90 are only weakly coupled to ATP hydrolysis. Nat. Struct. Mol. Biol. *16*, 281–286.

Mills, E.W., and Green, R. (2017). Ribosomopathies: There's strength in numbers. Science *358*, eaan2755.

Mills, E.W., Wangen, J., Green, R., and Ingolia, N.T. (2016). Dynamic Regulation of a Ribosome Rescue Pathway in Erythroid Cells and Platelets. Cell Rep. *17*, 1–10.

Mishra, S., Verhalen, B., Stein, R.A., Wen, P.C., Tajkhorshid, E., and Mchaourab, H.S. (2014). Conformational dynamics of the nucleotide binding

domains and the power stroke of a heterodimeric ABC transporter. eLife *3*, e02740.

Nir, E., Michalet, X., Hamadani, K.M., Laurence, T.A., Neuhauser, D., Kovchegov, Y., and Weiss, S. (2006). Shot-noise limited single-molecule FRET histograms: comparison between theory and experiments. J. Phys. Chem. B *110*, 22103–22124.

Nürenberg, E., and Tampé, R. (2013). Tying up loose ends: ribosome recycling in eukaryotes and archaea. Trends Biochem. Sci. *38*, 64–74.

Nürenberg-Goloub, E., Heinemann, H., Gerovac, M., and Tampé, R. (2018). Ribosome recycling is coordinated by processive events in two asymmetric ATP sites of ABCE1. Life Sci Alliance *1*, e201800095.

Pisarev, A.V., Skabkin, M.A., Pisareva, V.P., Skabkina, O.V., Rakotondrafara, A.M., Hentze, M.W., Hellen, C.U., and Pestova, T.V. (2010). The role of ABCE1 in eukaryotic posttermination ribosomal recycling. Mol. Cell *37*, 196–210.

Ploetz, E., Lerner, E., Husada, F., Roelfs, M., Chung, S., Hohlbein, J., Weiss, S., and Cordes, T. (2016). Förster resonance energy transfer and protein-induced fluorescence enhancement as synergetic multi-scale molecular rulers. Sci. Rep. *6*, 33257.

Rees, D.C., Johnson, E., and Lewinson, O. (2009). ABC transporters: the power to change. Nat. Rev. Mol. Cell Biol. *10*, 218–227.

Shao, S., Murray, J., Brown, A., Taunton, J., Ramakrishnan, V., and Hegde, R.S. (2016). Decoding Mammalian Ribosome-mRNA States by Translational GTPase Complexes. Cell *167*, 1229–1240.e15.

Shoemaker, C.J., and Green, R. (2011). Kinetic analysis reveals the ordered coupling of translation termination and ribosome recycling in yeast. Proc. Natl. Acad. Sci. USA *108*, E1392–E1398.

Smith, P.C., Karpowich, N., Millen, L., Moody, J.E., Rosen, J., Thomas, P.J., and Hunt, J.F. (2002). ATP binding to the motor domain from an ABC transporter drives formation of a nucleotide sandwich dimer. Mol. Cell *10*, 139–149.

Strunk, B.S., Novak, M.N., Young, C.L., and Karbstein, K. (2012). A translation-like cycle is a quality control checkpoint for maturing 40S ribosome subunits. Cell *150*, 111–121.

Thomas, C., and Tampé, R. (2018). Multifaceted structures and mechanisms of ABC transport systems in health and disease. Curr. Opin. Struct. Biol. *51*, 116–128.

van den Elzen, A.M., Schuller, A., Green, R., and Séraphin, B. (2014). Dom34-Hbs1 mediated dissociation of inactive 80S ribosomes promotes restart of translation after stress. EMBO J. *33*, 265–276.

van der Does, C., and Tampé, R. (2004). How do ABC transporters drive transport? Biol. Chem. *385*, 927–933.

Yang, M., Livnat Levanon, N., Acar, B., Aykac Fas, B., Masrati, G., Rose, J., Ben-Tal, N., Haliloglu, T., Zhao, Y., and Lewinson, O. (2018). Single-molecule probing of the conformational homogeneity of the ABC transporter BtuCD. Nat. Chem. Biol. *14*, 715–722.

Zimmerman, C., Klein, K.C., Kiser, P.K., Singh, A.R., Firestein, B.L., Riba, S.C., and Lingappa, J.R. (2002). Identification of a host protein essential for assembly of immature HIV-1 capsids. Nature *415*, 88–92.

Zutz, A., Hoffmann, J., Hellmich, U.A., Glaubitz, C., Ludwig, B., Brutschy, B., and Tampé, R. (2011). Asymmetric ATP hydrolysis cycle of the heterodimeric multidrug ABC transport complex TmrAB from *Thermus thermophilus*. J. Biol. Chem. *286*, 7104–7115.

STAR★METHODS

KEY RESOURCES TABLE

REAGENT or RESOURCE	SOURCE	IDENTIFIER
Antibodies		
Mouse monoclonal anti-polyhistidine	Sigma-Aldrich	Cat# A7058; RRID: AB_258326
Goat anti-mouse IgG	Sigma-Aldrich	Cat# AP124A; RRID: AB_92456
Bacterial and Virus Strains		
Cloning Strain One Shot Mach1 T1	Invitrogen	Cat# C862003
Expression strain BL21(DE3)- pRARE	Novagen	Cat# CMC0014
Sulfolobus solfataricus P2	Albers et al., 2003	DSM-1617
Thermococcus celer	Barthelme et al., 2011	DSM-2476
Chemicals, Peptides, and Recombinant Proteins		
AMP-PNP Lithium salt	Sigma-Aldrich	Cat# A2647
ATP	Sigma-Aldrich	Cat# A89337
[gamma] 32P-ATP	Hartmann Analytics	Cat# SPR-301
ADP	Sigma-Aldrich	Cat# A23831
ATTO647N-maleimide	Atto-TEC	Cat# AD647N-41
Cy3B-maleimide	GE Healthcare	Cat# PA63131
Alexa555 C2-maleimide	Invitrogen	Cat# A20346
Alexa647 C2-maleimide	Invitrogen	Cat# A20347
Dithiothreitol (DTT)	Carl-Roth	Cat# 4227.1
Carbenicillin	Carl-Roth	Cat# 6344.3
Chloramphenicol	Carl-Roth	Cat# 3886.1
Spermine	Carl-Roth	Cat# 7162.2
Isopropyl-β-D-thiogalactopyranoside (IPTG)	Carl-Roth	Cat# I6758
2-Mercaptoethanol	Carl-Roth	Cat# 4227.1
DNase I recombinant, RNase-free	Roche	Cat# 4716728001
RiboLock RNase inhibitor	ThermoFisher	Cat# EO0382
SulfoLink^{TM} Coupling Resin	ThermoFisher	Cat# 20401
Ni Sepharose^{TM} High Performance	Sigma-Aldrich	Cat# GE17-5268-01
Critical Commercial Assays		
NucleoSpin Plasmid EasyPure	Macherey-Nagel	Cat# 740727.10
Oligonucleotides		
ABCE1_I124C_fwd: CTAGCTGGTGAAATATGCCCAAATTTT GGAGATC	This paper	N/A
ABCE1_K177C_fwd: TTCAAAATTCCTTTGCGGTACGGTGAATG	This paper	N/A
ABCE1_T393C_fwd: GTTGGCGAAATTTGCGCAGATGAAGG	This paper	N/A
ABCE1_K430C_rev: GAAAGAGCGTCACAACTCGCATTT TCTAAGTATTG	This paper	N/A
Recombinant DNA		
Plasmid: pSA4_SsABCE1_wt	Barthelme et al., 2007	N/A
Plasmid: pSA4_SsABCE1_I124C/K430C	This paper	N/A
Plasmid: pSA4_SsABCE1_K177C/T393C	This paper	N/A

(Continued on next page)

Continued

REAGENT or RESOURCE	SOURCE	IDENTIFIER
Plasmid: pSA4_Ss.aRF1	Barthelme et al., 2011	N/A
Plasmid: pSA4_Ss.aPelota	Nürenberg-Goloub et al., 2018	N/A
Plasmid: pSA4_Ss.aIF6	Nürenberg-Goloub et al., 2018	N/A
Software and Algorithms		
Dual-Channel-Burst-Search	Nir et al., 2006	N/A
GraphPad Prism	GraphPad	RRID: SCR_002798
ImageJ	NIH	RRID: SCR_003070
LabView data acquisition	Nir et al., 2006	N/A
Mathematica	Wolfram	RRID: SCR_014448
MATLAB	MathWorks	RRID: SCR_001622
Origin	OriginLab	RRID: SCR_002815
PyMol	Schrödinger	RRID: SCR_000305
Other		
Amicon Ultra-15, Centrifugal Filters, 50 kDa	Merck Millipore	Cat# UFC905096
Amicon Ultra-15, Centrifugal Filters, 100 kDa	Merck Millipore	Cat# UFC910024

LEAD CONTACT AND MATERIALS AVAILABILITY

Further information and requests for resources and reagents should be directed to and will be fulfilled by the Lead Contact, Robert Tampé (tampe@em.uni-frankfurt.de).

EXPERIMENTAL MODEL AND SUBJECT DETAILS

Bacterial strains

E. coli strain One Shot Mach1 T1 (Invitrogen) for cloning of ABCE1 variants. *E. coli* strain BL21(DE3) (Novagen) transformed with pRARE plasmid (Novagen), coding for rare amino acids, was used for expression of ABCE1 variants and release factors.

METHOD DETAILS

Plasmids

$ABCE1^{wt}$ or $ABCE1^{\Delta FeS}$ from *S. solfataricus* were cloned with a C-terminal His_6-tag in pSA4 vector, which is based on a pET15b expression vector (Barthelme et al., 2011, 2007). Site-directed mutagenesis was used to construct double-cysteine variants of $ABCE1^{wt}$ or $ABCE1^{\Delta FeS}$ by megaprimer PCR. Plasmids were transformed into One Shot Mach1 T1 cells and purified using NucleoSpin Plasmid EasyPure kit (Macherey-Nagel) following the manufacturer's protocol. The identity and integrity of all ABCE1 variants were verified by sequencing. ABCE1 constructs, release factors and aIF6 were co-transformed with the pRARE plasmid (Novagen) coding for rare tRNAs into the BL21(DE3) *E. coli* strain (Novagen).

Protein purification

ABCE1 variants and aIF6 from *S. solfataricus* were transformed into BL21 (DE3) and expressed in terrific broth (TB) media (1.2% (w/v) peptone, 2.4% (w/v) yeast extract, 72 mM K_2HPO_4, 17 mM KH_2PO_4, and 0.4% (v/v) glycerol) supplemented with 100 μg/ml carbenicillin and 25 μg/ml chloramphenicol at 37°C, until an OD_{600} of 0.6 was reached. The temperature was lowered to 20°C and expression was induced after reaching an OD_{600} of 0.8 by adding 0.5 mM isopropyl-β-D-thiogalactopyranoside (IPTG). Cells were harvested 16–18 h after induction at 20°C. The aPelota and aRF1 constructs (Barthelme et al., 2011; Nürenberg-Goloub et al., 2018) were expressed in (BL21 DE3) and grown in LB-Lennox medium (5 g/l yeast extract, 10 g/l tryptone, 5 g/l NaCl) supplemented with 100 μg/ml carbenicillin and 25 μg/ml chloramphenicol at 37°C. At an OD_{600} of 0.6, expression was induced as mentioned above. The cells were harvested after 3 h of growth. ABCE1 and factors from *S. solfataricus* were purified as described in (Nürenberg-Goloub et al., 2018).

Ribosome purification

To isolate 30S ribosomes from *S. solfataricus,* a SulfoLink™ resin chromatography was performed as described (Leshin et al., 2010). SulfoLink™ Coupling Resin (ThermoFisher) was prepared following the manufacturer's protocol and equilibrated with binding buffer (20 mM HEPES-KOH pH 7.5, 5 mM $Mg(OAc)_2$, 60 mM NH_4Cl, 1 mM DTT). *S. solfataricus* cells were resuspended in buffer M (20 mM

HEPES-KOH pH 7.5, 500 mM KCl, 10 mM $MgCl_2$, 0.5 mM EDTA, 2 mM DTT, 1 mM PMSF, 1 μg RNase free DNase (ThermoFisher), 133 U/ml RiboLock RNase inhibitor (ThermoFisher), sonicated and centrifuged for 30 min at 30,000x g. The cleared lysate was added onto the column and incubated twice for 15 min on ice. The column was washed three times with binding buffer and elution was performed twice with 1.25 mL of elution buffer (20 mM HEPES-KOH pH 7.5, 10 mM $Mg(OAc)_2$, 500 mM NH_4Cl, 2 mM DTT). Ribosomes were pelleted through a glycerol cushion (20 mM HEPES-KOH pH 7.5, 10 mM $Mg(OAc)_2$, 500 mM KCl, 2 mM DTT, 50% (v/v) glycerol) at 100,000x g for 15 h at 4°C. Pellets were resuspended in 100 μl cushion buffer and separated by sucrose density gradient centrifugation (10%/30% (w/v) sucrose, 20 mM HEPES-KOH pH 7.5, 10 mM KCl, 1 mM $MgCl_2$) 14 h at 4°C and 50,000x g (SW41 rotor, Beckman Coulter). Gradients were fractionated from top to bottom (Piston Gradient Fractionator, Biocomp) recording the absorption at 254 nm. Fractions containing 30S or 50S were pooled and concentrated in HEPES buffer using an Amicon® Ultra centrifuge device (cut-off 100 kDa, Merck Millipore). Concentration of the ribosomes was determined using the absorption at 254 nm, 1 OD equals 120 pmol and 60 pmol of 30S or 50S subunit, respectively (Leshin et al., 2010).

70S ribosomes from *S. solfataricus* are very labile and dissociate during ribosome purification, even at high $MgCl_2$ concentrations (Londei et al., 1986). Therefore, an appropriate model has been established with 70S from *T. celer* being bound and split by ABCE1 (Barthelme et al., 2011; Nürenberg-Goloub et al., 2018). To purify 70S from *T. celer* two protocols were adapted (Becker et al., 2012; Coureux et al., 2016). *T. celer* cell pellets (provided by Harald Huber, Centre for Archaea & Microbiology, University of Regensburg) were resuspended in S30* buffer (10 mM HEPES-KOH pH 7.5, 60 mM NH_4OAc, 14 mM $MgCl_2$, 1 mM DTT) and lysed on ice by sonification. The lysate was cleared twice by centrifugation for 30 min at 30,000x g. Ribosomes were pelleted through a high salt cushion (1.1 M sucrose, 1 M NH_4OAc, S30 buffer) for 4 h at 170,000x g. The pellet was resuspended in TrB25 (56 mM Tris-HCl pH 8, 250 mM KOAc, 80 mM NH_4OAc, 25 mM $MgCl_2$, 1 mM DTT). 70S were separated from 30S and 50S with a 10%–40% linear sucrose gradient (10 mM HEPES-KOH pH 7.5, 60 mM NH_4OAc, 14 mM $MgCl_2$, 1 mM DTT) for 14 h at 68,000x g (SW41 rotor, Beckman Coulter). Gradients were fractionated as mentioned above. 70S containing fractions were pooled and concentrated with a Amicon® Ultra centrifuge device (cut-off 100 kDa, Merck Millipore) into S30* buffer. Concentration of 70S was determined as mentioned above.

Fluorescent labeling of ABCE1

10 nmol protein (50–100 μl volume, for biochemical studies 100 μM) in buffer A (50 mM Tris-HCl pH 7.2, 100 mM KCl, 5% (v/v) glycerol) were treated with 10 mM DTT for one hour on ice. DTT treated ABCE1 variant was diluted to 1 mL with buffer A and added immediately onto the equilibrated column material (Ni Sepharose^{TM} High Performance, GE Healthcare), incubated for 2 min and was subsequently washed twice with buffer A. To reach an ABCE1/Cy3B/Atto647N ratio of 1:10:8, Atto647N-maleimide (ATTO-TEC, 50 nmol aliquot) was dissolved in 5.5 μl DMSO (water-free), and 5 μl thereof were used to dissolve Cy3B-maleimide (GE Healthcare, 50 nmol aliquot). The dissolved dyes (2.5 μl) were diluted in 1 mL buffer A and added onto the resin and incubated agitating over night at 4°C shielded from light. The column was washed with 3 mL buffer A to remove excess of dyes. The protein was eluted with buffer B (500 μl; 50 mM Tris-HCl pH 7.2, 100 mM KCl, 250 mM imidazole, 5% (v/v) glycerol). Subsequently, a preparative gel filtration (Superdex 200 Increase PC 10/300; GE Healthcare) was carried in buffer C (50 mM Tris-HCl pH 8.0, 100 mM KCl) while recording the absorbance at 280 nm (protein), 559 nm (Cy3B), and 645 nm (Atto647N). Atto647N dye interacts non-specifically with the Superdex 200 column material allowing to enrich doubly-labeled ABCE1 variants (Husada et al., 2015). The labeling ratio was estimated according to ε_{ABCE1} = 71,220, ε_{Cy3B} = 130,000, and $\varepsilon_{Atto647N\,=}$ 150,000 (Barthelme et al., 2007).

Malachite Green ATPase assay

ATPase assays were performed to measure Michaelis-Menten kinetics of all double-cysteine variants with and without attached fluorophores. ATP hydrolysis was measured colorimetrically using a Malachite Green assay (Baykov et al., 1988). Triplicates of each reaction were measured in 25 μl ATPase buffer (20 mM Tris-HCl pH 7.5, 100 mM NaCl, 10 mM $MgCl_2$) with 5 μM of ABCE1 and 5 mM ATP. Reactions were incubated at 80°C for 10 min and stopped rapidly on ice by adding 175 μl of ice-cold stop solution (20 mM H_2SO_4). Complex formation was measured at 620 nm 10 min after addition of Malachite Green solution (3.5 mM Malachite Green, 0.18% (v/v) Tween-20, 1.15% (w/v) $(NH_4)_6Mo_7O_{24}$). The inorganic phosphate released was calculated with a standard curve. ATP hydrolysis by ABCE1 variants of three independent experiments was quantified by calculation of hydrolyzed ATP per min in Prism 5 (GraphPad).

Radioactive ATPase assay

ATPase activity of ABCE1 was further analyzed by the formation of $^{32}P_i$ upon hydrolysis of γ-^{32}P-labeled ATP as described in Heuer et al. (2017); Nürenberg-Goloub et al. (2018); Pisarev et al. (2010), and Shoemaker and Green (2011). 0.2 μM ABCE1 was incubated with 1 mM ATP and 0.5 μM [γ-^{32}P]ATP (Hartmann Analytics) or 0.5 μM *S. solfataricus* 30S and ATP in 20 μl of hot ATPase buffer (20 mM Tris-HCl pH 7.5, 100 mM KCl, 5 mM $MgCL_2$, 0.25 mM spermidine, 1 mM DTT) at 70°C. 1 μl was spotted after 0, 2, 5, 10 and 20 min onto polyethylene imine cellulose thin layer chromatography plates (Merck Millipore). Triplicates of every time point were spotted. The plates were resolved by 0.8 M LiCl and 0.8 M acetic acid. Release of $^{32}P_i$ was monitored by autoradiography (Typhoon 9400, GE Healthcare). Phosphoimages were quantified using ImageJ (NIH) and analyzed with Prism 5 (GraphPad). Probes

containing 30S and ABCE1 hydrolysis were background corrected (Nürenberg-Goloub et al., 2018). ATP hydrolysis was calculated from 20 min time point as mentioned above. SD was calculated from three independent experiments.

Formation of the pre- and post-splitting complex and ribosome splitting assay

To analyze ribosomal binding of non-labeled or fluorescently labeled ABCE1 double-cysteine mutants, 10%–30% (30S) or 10%–40% (70S) sucrose gradients were performed. Formation of the pre-splitting complex was probed by incubation of 1 μM of ABCE1 variants and 3 μM of purified T. celer 70S, 3 μM of aRF1 and aPelota and 2 mM of different nucleotides in 20 mM HEPES-KOH pH 7.5, 100 mM KCl, 50 mM $MgCl_2$, 2 mM DTT, 0.5 mM spermine. *T. celer* 70S were pre-incubated with aRF1/aPelota for 30 min at 25°C. Subsequently, ABCE1 and nucleotides were added and incubated for 1 h at 25°C. The post-splitting complex was probed by incubation of ABCE1 variants (3 μM) with purified 30S (4 μM) from *S. solfataricus* in presence of different nucleotides (2 mM) for 4 min at 73°C. Samples were loaded onto 10%–30% (30S) or 10%–40% (70S) (w/v) linear sucrose gradients. Centrifugation and fractionation were performed as mentioned above. 500 μl fractions were mixed with 1 mL acetone and precipitated over night at −20°C. Samples were either analyzed by SDS-PAGE combined with in-gel fluorescence (Typhoon 9400, GE Healthcare) or by immunoblotting using a monoclonal anti-His antibody (Sigma-Aldrich).

Splitting was analyzed by SDG centrifugation and the absorption at 254 nm as mentioned above. ABCE1 (2 μM) was incubated with *T. celer* 70S (0.5 μM), a mixture of the archaeal release factors aPelota and aRF1, the anti-reassociation factor aIF6 and AMP-PNP (0.125 μM) for 15 min at 45°C in splitting buffer (10 mM HEPES-KOH pH 7.5, 60 mM NH_4OAc, 14 mM $MgCl_2$) in a total volume of 50 μl. The reaction was cooled down on ice and loaded onto a 10%–40% (w/v) sucrose gradient in splitting buffer. Gradients were centrifuged 15 h at 68,000x g at 4°C (SW41 rotor, Beckman Coulter). Gradients were fractionated as mentioned above. The bars represent three independent experiments with a mean of the ratio of the 50S/70S peak heights. The bars are normalized to the splitting activity of wild-type ABCE1.

Sample preparation for smFRET and ALEX

ABCE1 variants (0.1–50 nM) were mixed with different binding partners and ligands such as 70S ribosomes, 30S ribosomal subunits or nucleotides. If not otherwise stated, saturating concentrations of 30S ribosomes (1 μM) and AMP-PNP/ADP (2 mM) were used. Binding reactions were performed in 20 mM HEPES-KOH pH 7.5, 200 mM KCl, 5 mM $MgCl_2$) at 73°C for indicated time periods (max. 10 min). By using ABCE1 from *S. solfataricus*, the reaction could be 'frozen' at any point in time during the process by cooling down the sample to 4°C with ice-cold cold buffer supplemented with TX and MEA (final concentration 1 mM TX, 10 mM MEA). A final ABCE1 concentration of 10–100 pM was reaced with by cooling down). 70S binding reactions with *T. celer* 70S (3 μM) were performed with aPelota and aRF1 (2 μM) and AMP-PNP (2 mM) in 20 mM HEPES-KOH pH 7.5, 100 mM KCl, 50 mM $MgCl_2$, 0.5 mM spermine, 1 mM DTT at 25°C for 1 h. To enrich the 70S bound fraction, the binding reaction was loaded onto a 10%–40% (w/v) stepwise sucrose gradient and centrifuged for 2.30 h at 220,000x g (MLS-50 rotor, Beckman Coulter). The gradients were fractionated in 200 μl fractions. 30S and 50S fractions were assigned by 254 nm absorption. The 30S and 70S fractions were diluted at least 2-fold in 70S binding buffer and subsequently used for single-molecule experiments.

To study the dissociation kinetics of 30S-bound ABCE1 we used two experimental approaches: (i) ADP competition or (ii) full removal of AMP-PNP by dilution and addition of unlabeled ABCE1 protein. (i) Labeled ABCE1 (30 nM) was incubated with AMP-PNP (10 mM, resembling intracellular ATP concentration) and 30S (12.5 μM) at 73°C for 10 min. After 630-fold dilution and addition of ADP (5 mM), the dissociation of the post-splitting complex was followed at 73°C for indicated time periods (0–10 min). (ii) Labeled ABCE1 (30 nM) was incubated with AMP-PNP (25 μM) and 30S (12.5 μM) at 73°C for 10 min. After 630-fold dilution by addition of unlabeled ABCE1 (2 μM final), the dissociation of the post-splitting complex was followed at 73°C for indicated time periods (0–10 min). A low (25 μM) AMP-PNP concentration is used which was sufficient for saturation of the closed state at both sites, as this can be efficiently sequestered after the dilution (40 nM) by 2 μM of unlabeled ABCE1 that yields no fluorescent background. Samples were subsequently analyzed with ALEX microscopy.

Anisotropy measurements and verification of the FRET-ruler character

Fluorescence spectra were derived on a standard scanning fluorometer (Jasco FP-8300; 20 nm excitation and emission bandwidth; 8 s integration time) and calculated at the emission maxima of the fluorophores (for Cy3B, $\lambda_{ex/em}$ 535/580 nm; for Atto647N, $\lambda_{ex/em}$ 635/660 nm), according to the relationship $R = (I_{VV} - GI_{VH})/(I_{VV} + 2GI_{VH})$. I_{VV} and I_{VH} describe the emission components relative to the vertical (V) or horizontal (H) orientation of the excitation and emission polarizer. The sensitivity of the spectrometer for different polarizations was corrected using horizontal excitation to obtain $G = I_{HV} / I_{HH}$. Typical G-values for Cy3B and ATTO647N were 0.65 and 0.43, respectively. We used 20 mM HEPES-KOH pH 7.5, 200 mM KCl, 5 mM $MgCl_2$ as a buffer and analyzed the anisotropy of the labeled protein and DNA samples in a concentration range of 50–2000 nM. The determined anisotropy values are summarized in Table S1; this includes a discussion on the FRET-ruler character of our assays.

Single-molecule fluorescence microscopy and ALEX

Single-molecule ALEX experiments were performed at room temperature (22°C) using a home-built confocal microscope similar to the described setup (Gouridis et al., 2015). In brief, two laser-diodes (Obis, Coherent, USA) with emission wavelength of 532 and 637 nm were directly modulated for alternating periods of 50 μs and used for confocal excitation. The laser beams where coupled

into a single-mode fiber (PM-S405-XP, Thorlabs, UK) and collimated (MB06, Q-Optics/Linos) before entering a water immersion objective (60X, NA 1.2, UPlanSAPO 60XO, Olympus). The fluorescence was collected by excitation at a depth of 20 μm. Average laser powers were 30 μW at 532 nm (~30 kW/cm^2) and 15 μW at 637 nm (~15 kW/cm^2). Excitation and emission were separated by a dichroic beam splitter (zt532/642rpc, AHF Analysentechnik), mounted in an inverse microscope body (IX71, Olympus). Emitted light was focused onto a 50 μm pinhole and spectrally separated (640DCXR, AHF Analysentechnik) on two APDs (τ-spad, < 50 dark-counts/s, Picoquant) with appropriate spectral filtering (donor channel: HC582/75; acceptor channel: Edge Basic 647LP; both AHF Analysentechnik). The signal was recorded using custom-written LabVIEW software (Kapanidis et al., 2004).

ALEX data analysis

The stoichiometry S and apparent FRET efficiency E* were calculated for fluorescent bursts having at least 250 photons, to yield a two-dimensional histogram (Kapanidis et al., 2004). Uncorrected FRET efficiency E* monitors the proximity between the two fluorophores via normalization of sensitized acceptor emission to the total fluorescence of both fluorophores during green excitation. S is defined as the ratio between the overall green fluorescence intensity over the total green and red fluorescence intensity and describes the ratio of donor-to-acceptor fluorophores in the sample. We used published procedures to identify bursts corresponding to single molecules (Eggeling et al., 2001). For this we used three parameters characterizing the burst: total of L photons with M neighboring photons within a time interval of T microseconds. For the data presented in this study, a dual-color burst search (Nir et al., 2006), using parameters M = 15, T = 500 μs and L = 25, was applied. Additional thresholding removed spurious changes in fluorescence intensity and selected for intense single-molecule bursts (all photons > 250 photons unless otherwise mentioned). E* and S values for each burst and thus for individual molecules were binned into a two-dimensional histogram, where we selected donor-acceptor-containing sub-populations according to their intermediate S values (see Figures S2A and S2B). The one-dimensional E* histograms were fitted with a mixture model of a variable number of Gaussian distributions (1-3). In the fitting procedure the mean and the amplitude were derived from fitting, whereas the standard deviation was fixed or allowed to vary over a small region defined from static DNA samples having attached fluorophores at specific positions (see Table S2). We used the minimum number of distributions that fitted the experimental data, in which the mean value defines the apparent FRET value (E*) and the amplitude the abundance of a conformational state. Custom-made software was applied (Gouridis et al., 2015; Ploetz et al., 2016). The errors of the populations were obtained by the SD from 3–5 measurements.

To remove unwanted signals from large fluorescent aggregates that generate a sequence of apparent bursts with similar but ambiguous (high) E values, we applied an additional filter based on the correlation between the bursts. To exclude an undesirable systematic influence of the filter on the relative distributions between open/intermediate/closed states, we analyzed all datasets with and without this filter and obtained identical percentages. In brief, X_i is a Bernoulli-distributed random variable specifying the E state of the *i*th burst with an E-value of E_i with its related probability p'. We define $X_i = 0$ for $E_i < e$ and $X_i = 1$ for $E_i > e$. An estimator for p' is the fraction of bursts in the dataset with E > e and is denoted by $\widehat{p}'$. Second, we define another random variable of the *i*th burst by $T_i = \sum_{j=1}^{n} X_{i-j} + X_{i+j}$. T_i has a binominal distribution with parameters 2n and p_i. An estimator for p_i is $\widehat{p}_i = (2n)^{-1}\sum_{j=1}^{n} X_{i-j} + X_{i+j}$. If all bursts in a given dataset are uncorrelated then $p_i = p'$. Since the observed fluorescent aggregates show unrealistically high FRET efficiencies we have $p_i > p'$. We designed a hypothesis test that probes whether the *i*th bursts with $X_i = 1$ comes from a single labeled fluorescent molecule or from a fluorescent aggregate. The hypothesis test is H_0: $p_i = p'$ versus H_1: $p_i > p'$. We set the critical region of acceptance of H_0 from $\widehat{p}_i = 0$ to $\widehat{p}_i = \widehat{p}' + w\sqrt{\widehat{p}'(1-\widehat{p}')}$. If for the *i*th burst $X_i = 1$ and H_0 is rejected, the burst is taken out from further analysis. To control type II errors, we chose the w-values between 0.5 and 2, depending on the relative amount of aggregation present. The large fluorescent aggregates create around 6-12 (correlated) bursts with an E-value of E > 0.9. To only select the bursts from aggregates, we chose n = 3 and e = 0.9. If no aggregation exists, the filter removes only 1%–4% of the data. In case there is an aggregation, 10%–15% of the data is filtered out.

To directly probe association of fluorescently-labeled ABCE1 to the 30S or 70S ribosome, we used the large increase in molecular mass and associated slower diffusion as an independent observable. For this purpose, a histogram for the size of the bursts was constructed with a bin-size of Δ (200–400 μs, depending of the amount of data). Let m_i denote the number of bursts having a burst-size between $i\Delta$-$\Delta/2$ and $i\Delta$+$\Delta/2$. The tail of the burst-size histogram can be approximated by the function:

$$P(t;D) = NDe^{-Dt} \tag{1}$$

where $t = i\Delta$, D is the relative diffusion constant and is proportional to the (translational) diffusion coefficient and the size of the excitation volume of the confocal microscope, and N is a constant that depends on the size of the excitation volume and is proportional to the number of data points in the histogram (Ko et al., 1997). We analyzed the burst-size histogram from $t_1 = n\Delta$ to $t_2 = m\Delta$, where $k = m - n + 1$ and used simple linear regression to obtain an estimator for D, denoted by $\widehat{D}$:

$$\widehat{D} = \frac{\sum_n^m \left(k^{-1}\sum_n^m \log m_i - \log m_i\right)\left(\Delta i - k^{-1}\sum_n^m \Delta i\right)}{\sum_n^m \left(\Delta i - k^{-1}\sum_n^m \Delta i\right)^2} \tag{2}$$

Note that, $-\widehat{D}$ is the *linear slope* of the histogram on the log-scale. Typically, $t_1 = 2$ ms and $t_2 = 8$ ms. The estimated relative diffusion constants of the 30S/70S bound ABCE1 and free ABCE1 are denoted by $\widehat{D_1}$ and $\widehat{D_2}$, respectively. The tail of the burst-size histogram of a mixture of 30S/70S-bound and free-ABCE1 can then be approximated by the function:

$$P(t; D_1, D_2, A) = N\left(A\, D_1\, e^{-D_1\, t} + (1 - A) D_2\, e^{-D_2\, t}\right) \tag{3}$$

where A is the fraction of ABCE1 molecules that are bound to 30S/70S ($D1$), and 1-A is the fraction of ABCE1 molecules that are free ($D2$). Contrary to Equation 1, Equation 3 is not linear on the log-scale. However, we can still calculate the *linear slope* $-\widehat{D}$ by using Equation 2. An estimator for A, denoted by $\widehat{A}$, was found by solving,

$$\widehat{D} = \frac{\sum_n^m \left(k^{-1} \sum_n^m \log P\left(\Delta i; \widehat{D_1}, \widehat{D_2}, \widehat{A}\right) - \log P\left(\Delta i; \widehat{D_1}, \widehat{D_2}, \widehat{A}\right)\right)\left(\Delta i - k^{-1} \sum_n^m \Delta i\right)}{\sum_n^m \left(\Delta i - k^{-1} \sum_n^m \Delta i\right)^2} \tag{4}$$

Importantly, due to the log-transform, the unknown constant N cancels in Equation 4, therefore allowing to obtain an estimator for A. All calculations were done with the software package *Mathematica* (Wolfram).

Confocal scanning microscopy and data analysis

To gain information on possible conformational sampling of ABCE1 at room temperature, we used the same home-built confocal microscope as described before (Gouridis et al., 2015). Surface scanning was performed using a XYZ-piezo stage with 100 × 100 × 20 μm range (P-517-3CD with E-725.3CDA, Physik Instrumente). The detector signal was registered using a Hydra Harp 400 picosecond event timer and a module for time-correlated single photon counting (both Picoquant). The data, e.g., time traces and scanning images, were extracted using custom made software. Data were recorded with constant 532-nm excitation at an intensity of 0.5 μW (~125 W/cm^2). Surface immobilization was conducted using an anti-His antibody and established surface-chemistry protocols as described (Gouridis et al., 2015).

QUANTIFICATION AND STATISTICAL ANALYSIS

Statistical analyses were performed with Origin software version 2018 (OriginLab), MATLAB R2014b (MathWorks) and GraphPad Prism 5 (GraphPad). The standard deviation in the ATPase assays in Figure 1D and Figure S1B and ribosome splitting data in Figure S1F were derived from the independent assays with labeled and unlabeled protein. FRET histograms were fitted with a Gaussian mixture model with a restricted standard deviation (see the STAR Methods section for details). The data (means and the amplitudes) correspond to mean ± SD of 3-5 repeated experiments (i.e., independent protein purification and labeled sample). The mean ± SD are indicated in Figures 3B, 3C, 4B, 4C, 4F, S2F, S3A, S3B, S4E, and S5B and Table S2. Diffusion data presented in Figures 2B, 2D, 4D, S2C, and S4C correspond to 95% confidence interval of the slope of a single measurement. Qualitatively consistent results were obtained upon repetition with an independently purified and labeled protein sample. Anisotropy values as presented in Table S1 correspond to mean ± SD of duplicate measurements with the same labeled sample.

DATA AND CODE AVAILABILITY

Data obtained in this study are included in the manuscript and the Supplemental Information. SmFRET histograms and burst size analysis are included in the manuscript. Primers used for generation of cysteine variants are listed in the Key Resources Table.

Cell Reports, Volume *28*

Supplemental Information

ABCE1 Controls Ribosome Recycling by an Asymmetric Dynamic Conformational Equilibrium

Giorgos Gouridis, Bianca Hetzert, Kristin Kiosze-Becker, Marijn de Boer, Holger Heinemann, Elina Nürenberg-Goloub, Thorben Cordes, and Robert Tampé

Supplemental Items

	Anisotropy R	
	Cy3B	Atto647N
Free dye	0.08 ± 0.01	0.05 ± 0.01
ds22 ruler (dsDNA/Cy3B-8bp-Atto647N)	0.13 ± 0.02	0.16 ± 0.03
ds24 ruler (dsDNA/Cy3B-18bp-Atto647N)	0.15 ± 0.03	0.17 ± 0.04
ABCE1$^{K177C/T393C}$	0.20 ± 0.03	0.22 ± 0.05
ABCE1$^{I124C/K430C}$	0.21 ± 0.04	0.23 ± 0.05

Table S1. Anisotropy values derived from ensemble measurements, Related to Figure 1 and Experimental Methods

We applied a similar strategy as described before that uses steady-state anisotropy to verify that the measurements of E* report on relative differences in inter-probe distances of donor and acceptor molecules (Gouridis et al., 2015). In this procedure, steady-state anisotropy values of protein samples and static references based on double-stranded DNA (45-bp oligonucleotides) labeled with Cy3B and Atto647N (IBA, Germany) were compared. In the dsDNA system, an increase in the separation between donor and acceptor, results in decreasing FRET efficiency, which experimentally verifies that FRET serves as a nanoscopic ruler. For DNA the anisotropy was <0.17 and <0.23 for ABCE1 (Table S1). We used a published theory (Haas et al., 1978) to estimate the relative error associated with distance determination in both dsDNA and ABCE1 when erroneously assuming a fixed dye orientation (κ2~ 2/3). Haas and co-workers provide this error as ratio r/r' of true distance r to apparent distance r'; this ratio (=uncertainty) is moderate for anisotropies r<0.3 of both dyes (Haas et al., 1978). We find r/r' <12% for dsDNA and <20% r/r' for ABCE1 variants. These results and considerations confirm that FRET can act as a nanoscopic ruler in our assays. Additional measurements of site II variant labeled with a different dye pair (Alexa555/Alexa647, ThermoFisher Scientific) confirm the three distinct conformational states at E* = 0.56 (low FRET), E* = 0.69 (intermediate FRET), E* = 0.86 (high FRET) at 50 nM of 30S, 2 mM AMP-PNP and 10 min incubation at 73 °C resembling conditions shown in Figure 3. Although the protein values are slightly elevated compared to dsDNA samples, additional control experiments with a different dye pair on ABCE1 confirm the occurrence of three different FRET states in ABCE1 with low, intermediate and high FRET efficiency as given in **Supplemental Table S2**.

	low FRET (open state)	intermediate FRET (intermediate state)	high FRET (closed state)
Site I (Cy3B/Atto647N) in free ABCE1	0.55 ± 0.04	0.64 ± 0.04	0.79 ± 0.03
Site I (Cy3B/Atto647N) in pre-SC	0.53 ± 0.07	0.65 ± 0.03	0.78 ± 0.08
Site I (Cy3B/Atto647N) in post-SC	0.50 ± 0.01	0.61 ± 0.03	0.76 ± 0.04
Site II (Cy3B/Atto647N) in free ABCE1	0.47 ± 0.02	0.60 ± 0.01	–
Site II (Cy3B/Atto647N) in pre-SC	0.49 ± 0.06	0.62 ± 0.08	0.83 ± 0.03
Site II (Cy3B/Atto647N) in post-SC	0.51 ± 0.01	0.62 ± 0.03	0.80 ± 0.05
ΔFeS Site II (Cy3B/Atto647N) in free ABCE1	–	0.61 ± 0.02	0.79 ± 0.03
ΔFeS Site I (Cy3B/Atto647N) in post-SC	0.50 ± 0.01	0.61 ± 0.02	0.75 ± 0.01
ΔFeS Site II (Cy3B/Atto647N) in post-SC	–	0.61 ± 0.01	–
Site II (Alexa555/Alexa647) in post-SC	0.56 ± 0.02	0.69 ± 0.03	0.86 ± 0.03
ds22 (dsDNA/Cy3B-8bp-Atto647N)	0.90 ± 0.04 (0.066 ± 0.001)		
ds23 (dsDNA/Cy3B-13bp-Atto647N)	0.64 ± 0.01 (0.078 ± 0.001)		
ds24 (dsDNA/Cy3B-18bp-Atto647N)	0.45 ± 0.02 (0.076 ± 0.001)		
ds27 (dsDNA/Cy3B-33bp-Atto647N)	0.17 ± 0.03 (0.061 ± 0.002)		

Table S2. Apparent FRET Values of dsDNA Standards and ABCE1 Variants with Different Labels, Related to Figure 1, 2 and Experimental Methods

It is important to note that absolute FRET values (E*) reported in this manuscript cannot be directly compared since they were not corrected for spectral crosstalk, differences in donor and acceptor fluorescence quantum yield, and different detection efficiencies of the donor and acceptor channels. Since the second tested dye-pair Alexa555/Alexa647 further has a distinct Förster radius of R_0 = 5.1 nm from Cy3B/Atto647N (R_0 = 5.5–6.5 nm) (Ploetz et al., 2016), different FRET values were obtained for 70S- and 30S-bound ABCE1. As additional controls we report mean E* and sigma-values of experiments using double-stranded DNA with fluorophores at 8 bp, 13 bp, 18 bp, or 33 bp separation. These gave us the possibility to define the standard deviation (reported into parenthesis) for static samples with different apparent FRET values (E*). The width of the distribution (standard deviation) is a variable in smFRET ALEX experiments that is (largely) independent of the molecular details of the sample, but depends on the (limited) number of photons obtained from single-molecules. The more photons that are detected from a single-molecule the smaller the standard deviation and *vice versa* (Nir et al., 2006).

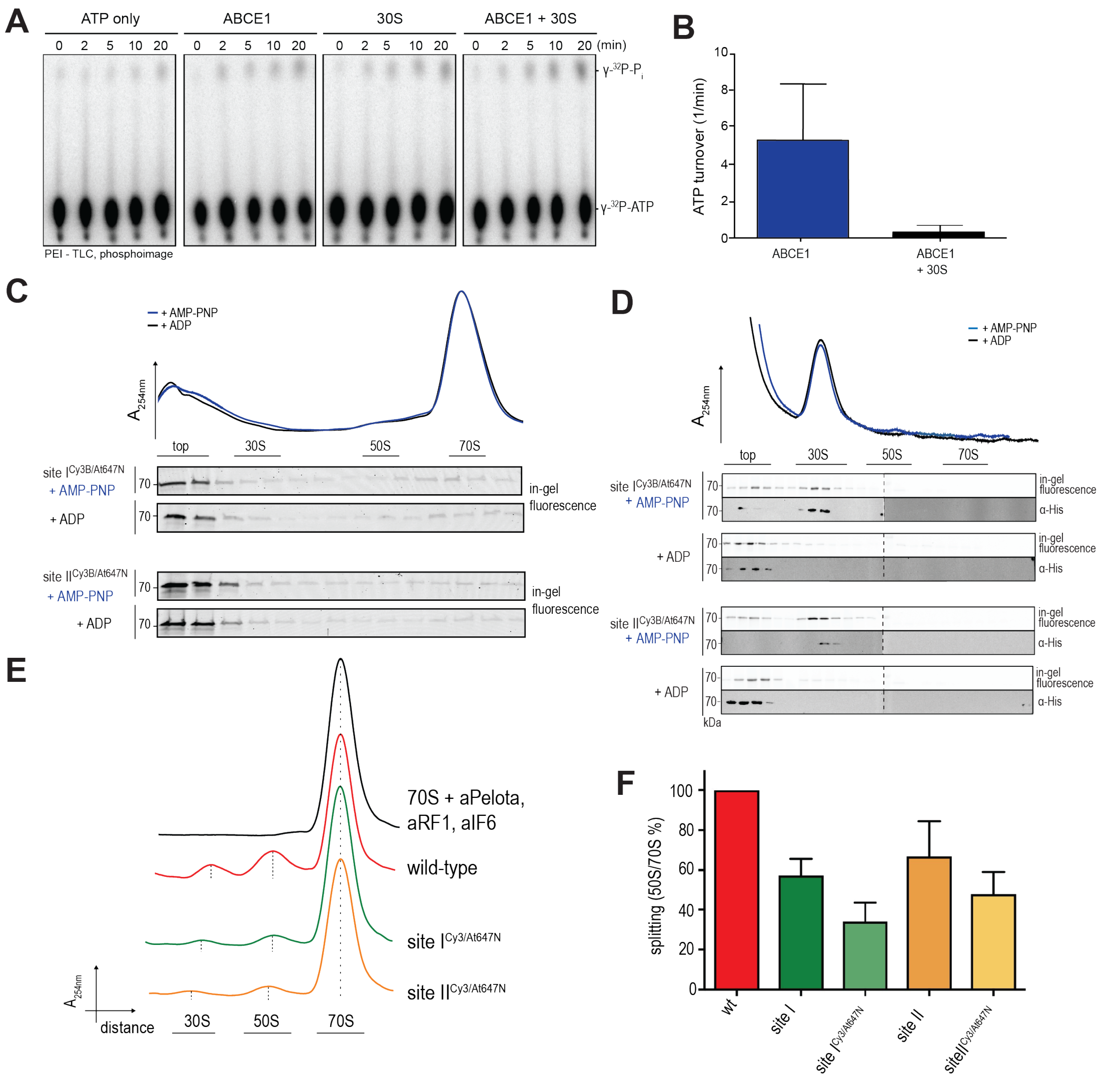

Figure S1. Formation of the Pre- and Post-SC with FRET Pair Labeled ABCE1, Related to Figure 1.

(A) Hydrolysis of γ-^{32}P-labeled ATP resulted in the formation of γ-$^{32}P_i$, which was separated by thin layer chromatography and subsequently quantified by autoradiography. Time course over 20 min. (B) ABCE1 shows no ATPase activity upon addition of 30S ribosomes. Hydrolysis of ABCE1 and ABCE1+30S were corrected for auto-hydrolysis and ABCE1+30S corrected for hydrolysis of 30S only. ATP turnover was calculated from 20 min point of time. Data derived from (B) are given as mean ± SD from three experiments. (C) AMP-PNP- and aRF1/aPelota-dependent formation of the pre-SC. A-site factors aRF1/aPelota (3 µM) and *T. celer* 70S ribosomes (3 µM) were pre-incubated for 30 min at 25 °C. Subsequently, ABCE1 (1 µM) and AMP-PNP (2 mM) were added and incubated for 1 h at 25 °C to form the complex. The specific binding of labeled site II variant to 70S ribosomes was analyzed by sucrose density centrifugation and subsequent in-gel fluorescence. (D) AMP-PNP-dependent formation of the post-SC. ABCE1 (3 µM) was incubated with 30S ribosomes (4 µM) for 10 min at 73 °C to form the post-SC. The specific binding of labeled ABCE1 variants to 30S ribosomes was analyzed by sucrose density centrifugation and subsequent in-gel fluorescence and immunoblotting. (E) Splitting of 70S (0.5 µM) by wild-type ABCE1 and site I and II variants (2 µM) with AMP-PNP (0.125 mM), aRF1/aPelota and the anti-association factor aIF6 for 15 min at 45 °C. (F) Splitting efficiency of wtABCE1 and site I and II variants. 50S/70S ratio was calculated by the peak height. Since at least 85% of ABCE1 molecules are doubly labelled, having correctly assembled FeS clusters and display a monodisperse population in size-exclusion chromatography (see text for details), we conclude that the reduction in splitting efficiency depends mainly on the cysteine mutation and minimally on fluorophore attachment. Clearly, the labelled cysteine variants (site I and II) retain high degree of functionality. The bars are normalized to the splitting activity of wtABCE1. Data represent mean ± SD from three measurements.

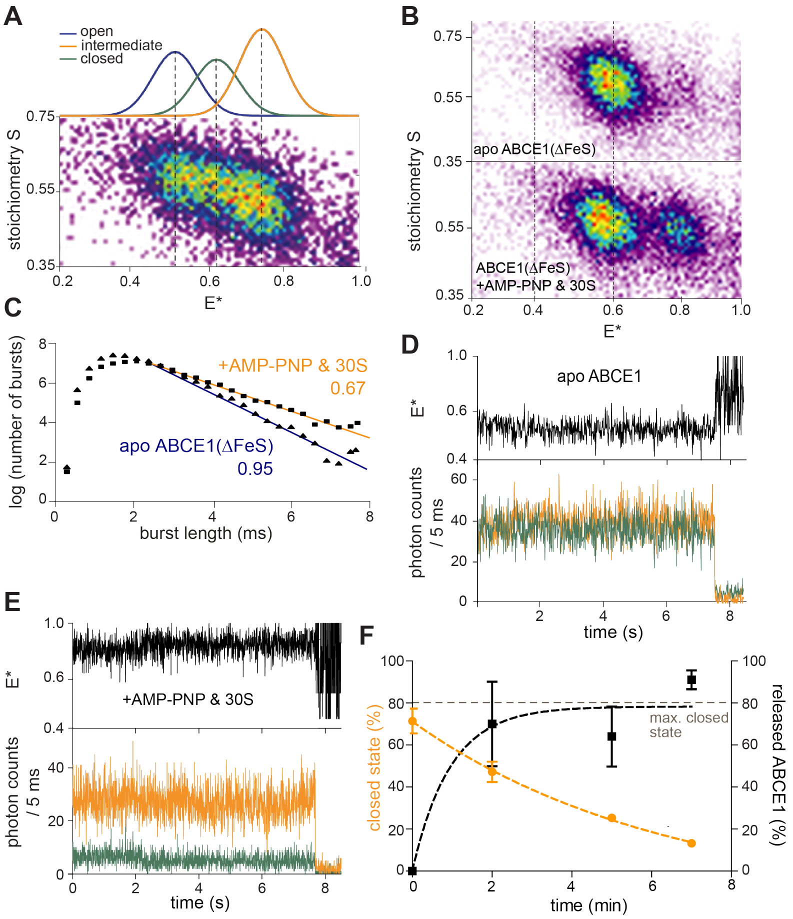

Figure S2. Conformational States of site I, ΔFeS site II and I Variant Compared to Full-length ABCE1, Related to Figure 2 and 3.

(A) 2D-ALEX histogram of site I after incubation with non-saturating concentration of 30S ribosomal subunit (25 nM) and AMPPNP (2 mM) for 10 min at 73 °C. Site I resembles an equilibrium between an open (blue, E*= 0.5), intermediate (green, E*= 0.61) and closed sate (orange, E*= 0,76). (B) 2D-ALEX analysis of ΔFeS site II variant labeled with Cy3B and Atto647N. Two different conformational states of ΔFeS site II (intermediate and closed) are observed. (C) 30S in complex with ABCE1 was investigated via an exponential fit of the burst-length distribution that indirectly probes molecular diffusion times of labeled ABCE1. The data show that also ΔFeS site II variant can bind to the ribosome and adopt the closed state. The derivation of a burst-size histogram from all molecules of one condition (apo vs. post-SC) shows that ΔFeS site II variant has reduced diffusion speed (0.67) and a small percentage of closed state (E* = 0.79) under post-splitting conditions when in complex with 30S and AMP-PNP. The intermediate value of the relative diffusion coefficient of post-SC conditions (0.67, compared to 0.53 of site II) indicates that only a subpopulation of all molecules is in complex with 30S. Specifically, we note that ABCE1 in the closed conformation (E* > 0.7) is completely 30S bound, whereas in the intermediate conformation (E* < 0.7) only a small subpopulation of ABCE1 molecules present is associated to 30S. These results demonstrate that the FeS cluster domain is not essential for conformational changes of site II or 30S-ABCE1 interaction. Nevertheless, the FeS cluster domain is essential for ribosome splitting and stable 30S association (Barthelme et al., 2011; Karcher et al., 2008). This observation is fully compatible with the fact that the closed state has the highest affinity for the 30S subunit. Since the ΔFeS site II variant-30S interaction is of short-lived nature (see also **Figure S3A/B**, compare bars 5 to 6 for ΔFeS site II or 4 to 5 for ΔFeS site II), this association could not be observed in previous experiments using sucrose density gradient centrifugation (Barthelme et al., 2011). The FeS cluster domain is required for dynamics in site II and stable ribosome interaction (Barthelme et al., 2011; Karcher et al., 2008). (D and E) Fluorescent trajectories of the immobilized site II derivative using confocal scanning microscopy analysis demonstrate the static nature of the apo (D) and the post-splitting state (E) of ABCE1 at room-temperature on the 10 s time scale. (F) The release of ABCE1 from the small ribosomal subunit (black) and its interdependence with the site I opening (orange) is depicted. Data represent mean ± SD from three measurements.

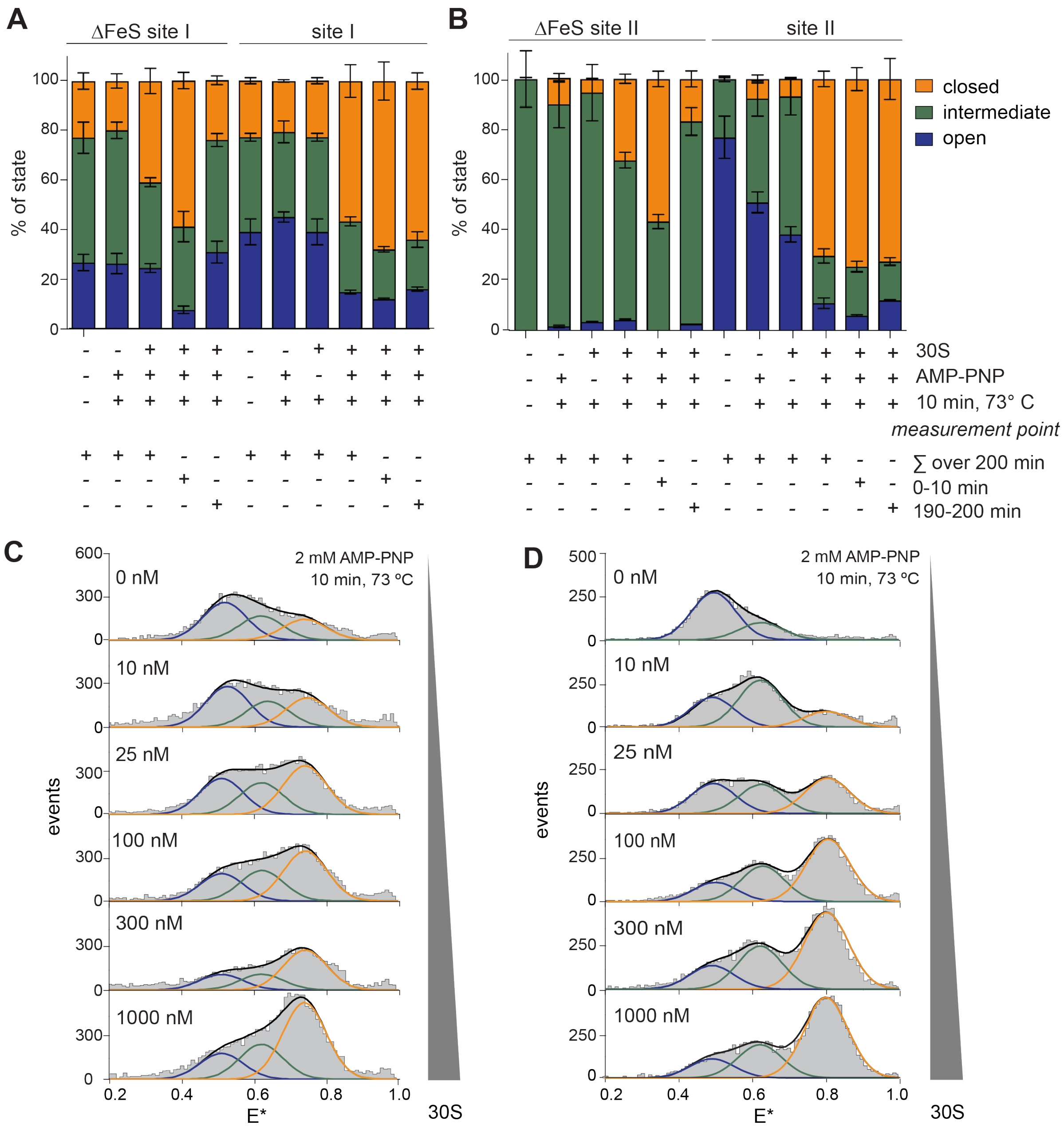

Figure S3. Conformational States of ΔFeS site II and I Variant in Comparison to Full-length Variants, Related to Figure 2, 3 and 4.

(A and B) Conformational states of ΔFeS and full-length site I and II variants under various conditions. Each measurement was performed under saturating conditions with 1 μM 30S ribosomal subunit and 2 mM nucleotide where indicated. Data represent mean ± SD from three measurements. (C and D) Conformational dynamics at site I (C) and site II (D) are probed in dependence of increasing 30S ribosomal subunit concentrations at saturating AMP-PNP concentrations (2 mM).

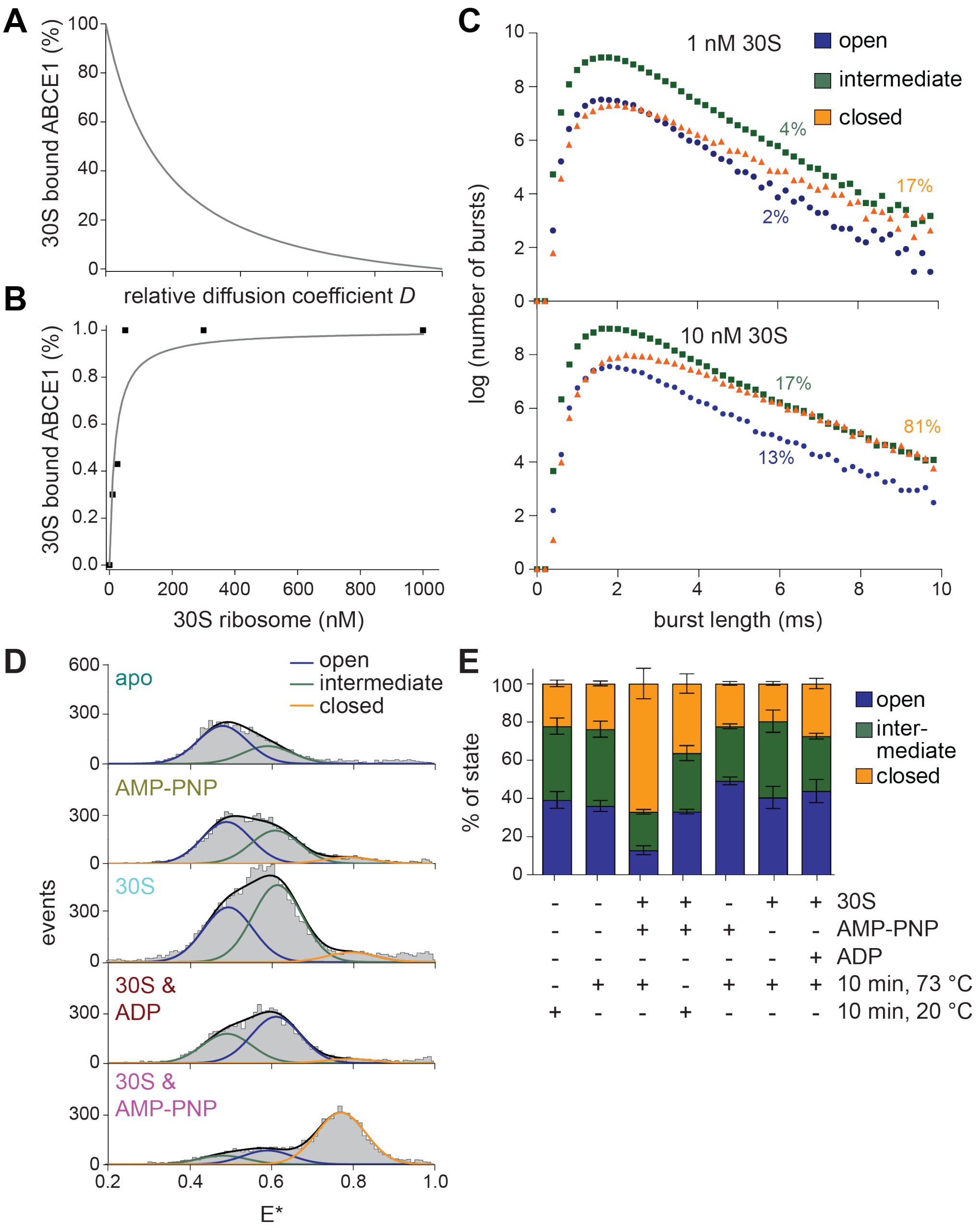

Figure S4. Different 30S Binding Affinities for the Open, Intermediate, and Closed State of ABCE1, Related to Figure 4.

(A) Relation of determined relative diffusion coefficient D (slope from exponential fits of burst-size histogram as shown in panel (C) to % of 30S-bound ABCE1 molecules (details see Materials and Methods). (B) Direct determination of 30S binding affinity for ABCE1 in the presence of 2 mM AMP-PNP using the relation between relative diffusion coefficient and 30S-bound % applying the data presented in Figure 4A and Figure S3C/D. Obtained K_D value of ~ 20 nM. (C) Experimental data to determine relative diffusion times for different ABCE1 conformational states. Here, the burst-length distribution of each FRET population (low, intermediate, high) was analyzed to derive a relative diffusion coefficient. The data shows that the high FRET state (closed) has the highest affinity since it is being >80% bound to 30S ribosome when increasing 30S from 1 nM to 10 nM, while low and intermediate FRET are still < 20% bound to 30S. (D and E) Influence of different ligands such as nucleotides and ribosomal subunits on the conformational states of site II (D) and site I (E). The experiments were performed under saturation conditions of nucleotides (2 mM) and 30S ribosomal subunits (1 µM) after 10 min incubation at 73 °C as indicated in the respective panel (D and E). Data in (E) represent mean ± SD from 3-5 measurements.

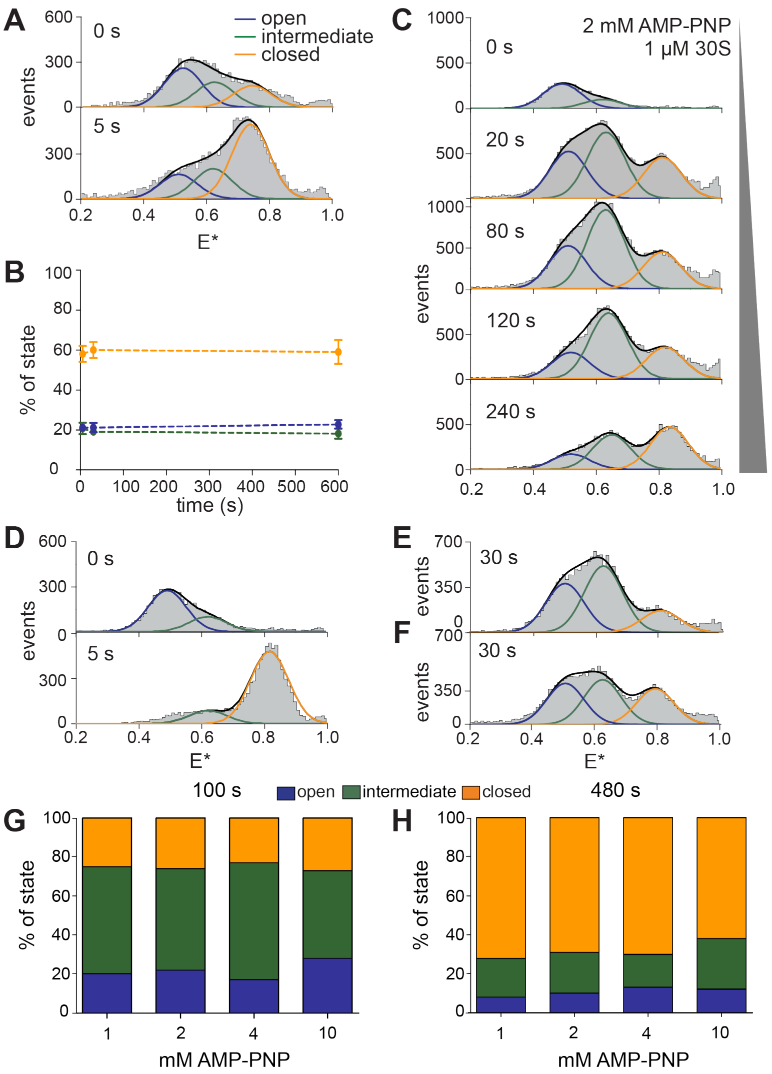

Figure S5. Elucidation of the Rate-limiting Step for Conformational Changes in Site I and the Time-dependence of the Conformational Changes in Site II, Related to Figure 4.

(A and B) The rate-determining step of conformational dynamics at site I was addressed by pre-incubated ABCE1 site I variant with AMP-PNP (2 mM) for 10 min at 73 °C (A, top panel). Subsequently, 30S subunit (1 µM) was added and confocal ALEX measurements were performed to monitor the state distribution after 5 s (A, bottom panel). Confocal ALEX measurements (A) and the changes in the distribution of the three states in site I are shown as a function of time after AMP-PNP pre-incubation (B). Data represent mean ± SD from three measurements. (C) Time dependent conformational dynamics at site II probed at saturating 30S ribosomal subunit (1 µM) and AMP-PNP concentrations (2 mM) at the indicated time points. (D-F) The rate-determining step of conformational dynamics at site II were addressed by use of pre-incubated ABCE1 site II variant either with AMP-PNP (2 mM, D), 30S (1 mM, E), or solely incubation at 73 °C (F). After incubation for 10 min at 73 °C, 30S (1 µM, D and F) or AMP-PNP (2 mM, E) were added, respectively, and confocal ALEX measurements were performed. Data represent mean ± SD from 3-5 measurements. (E and F) Controls where the pre-incubation was performed with 30S (1 mM, E) or heat only (10 min, 73 °C, F). (G and H) Conformational dynamics at site II as described for (D-F) and Fig. 4E. Pre-incubation was accomplished for 100 s (G) and 480 s (H) with 1-10 mM of AMP-PNP as the intracellular ATP concentration ranges from 1 to 10 mM and ATP hydrolysis obeys Michaelis-Menten kinetics with $K_{m,ATP} \sim 0.7$ mM. Increasing the ATP concentration from 1-10 mM increases the association rate by one order of magnitude, as this linearly correlates with the concentration. The kinetics to reach the conformational equilibria are independent of AMP-PNP concentration and require a long incubation subsequent to AMP-PNP binding.

Supplemental References:

Haas, E., Katchalski-Katzir, E., and Steinberg, I.Z. (1978). Effect of the orientation of donor and acceptor on the probability of energy transfer involving electronic transitions of mixed polarization. Biochemistry *17*, 5064-5070.
